# Comprehensive Analysis of the AP2/ERF Transcription Factor Family in *Schisandra chinensis* and Its Implications for secondary metabolism

**DOI:** 10.1371/journal.pone.0361017

**Published:** 2026-10-15

**Authors:** Kai Qian, Yifen Huang, Pengfei Zhu, Jing Wan, Jun Cheng, Zhuohang Li, Wensen Xie, Chengying Shan, Bin Chen, Yusong Shen, Wei Nie

**Affiliations:** 1 Jiangsu Product Quality Testing & Inspection Institute, Nanjing, China; 2 Fujian Pien Tze Huang Cosmetics Co., Ltd., Zhangzhou, Fujian, China; 3 Nanjing Research Institute for Comprehensive Utilization of Wild Plants, Nanjing, China; ICAR - National Institute for Plant Biotechnology, INDIA

## Abstract

The AP2/ERF transcription factor superfamily is integral to plant secondary metabolism; however, its comprehensive genomic characterization in *Schisandra chinensis*—a medicinal plant esteemed for its dibenzocyclooctadiene (DBCOD) lignans with a wide range of pharmacological properties—has not yet been conducted. In this study, we identified a total of 144 *ScAP2/ERF* genes from the high-quality, chromosome-level genome of *S. chinensis*. These genes were categorized into five subfamilies (ERF, DREB, AP2, RAV, and Soloist) through phylogenetic analysis with *Arabidopsis thaliana*. Conserved motif analysis confirmed the presence of the characteristic AP2 domain in all ScAP2/ERF proteins, with members of the AP2 subfamily containing two AP2 domains and RAV members featuring both AP2 and B3 domains. Chromosomal localization analysis revealed an uneven distribution of these genes across 14 chromosomes, with clusters identified on chromosomes 1, 2, 6, 10, 11, and 13. Cis-acting element analysis demonstrated an enrichment of light-responsive, MeJA-responsive, ABA-responsive, and anaerobic induction elements in the promoters of *ScAP2/ERF* genes. Synteny analysis identified 26 pairs of segmentally duplicated genes within the *S. chinensis* genome and 39 orthologous pairs with *A. thaliana*. Expression profiling across roots, stems, leaves, and fruits revealed distinct tissue-specific expression patterns. Of the 144 identified genes, 122 (84.7%) were detectably expressed in at least one tissue; among them, ten exhibited notably high expression in fruit tissues, suggesting their potential regulatory roles in the accumulation of pharmacologically active compounds in the medicinal fruit. This study represents a comprehensive genome-wide identification of the AP2/ERF family in *S. chinensis*, thereby establishing a foundational framework for understanding its evolutionary history and functional diversification. Furthermore, it offers candidate genes for subsequent functional validation in the regulation of their potential roles in regulating secondary metabolism.

## Introduction

*Schisandra chinensis* (Turcz.) Baill., belonging to the family Magnoliaceae, produces dried mature fruits that are used as a medicinal material, commonly known as “Bei Wuweizi”, and can also be consumed as food [1]. This herb has been used in traditional Chinese medicine for over two millennia [2]. Modern pharmacological studies have confirmed that extracts of *S. chinensis* display significant antioxidant, anti-inflammatory, neuroprotective, hepatoprotective, antidiabetic, antidepressant, and gut microbiota‑regulating activities [3–5]. Its broad pharmacological actions are attributed to its abundant chemical constituents, such as lignans, triterpenoids, polysaccharides, and volatile oils [6]. Among these, lignans are the major bioactive compounds, belonging to dibenzocyclooctadiene (DBCOD) lignans, representative examples including schisandrol A, gomisin A, and schisandrin A [7]. The biosynthetic pathway of DBCOD lignans has been progressively elucidated, involving key enzymes such as phenylalanine ammonia-lyase (PAL), cinnamate 4-hydroxylase (C4H), 4-coumarate-CoA ligase (4CL), cinnamoyl-CoA reductase (CCR), cinnamyl alcohol dehydrogenase (CAD), dirigent protein (DIR), and various P450 enzymes and O-methyltransferases [8–10].

The AP2/ERF transcription factor superfamily, known as APETALA2/Ethylene-Responsive Factor, is found throughout the plant kingdom and plays a role in stress responses, hormone signaling, and the production of various secondary metabolites [11]. In *Arabidopsis thaliana*, the AP2/ERF family is classified into five subfamilies: AP2, ERF, DREB, RAV, and Soloist [12]. Importantly, accumulating evidence has demonstrated that AP2/ERF transcription factors serve as key regulators of specialized metabolic pathways across multiple medicinal plant species. For instance, *SmERF1L1* directly activates tanshinone biosynthetic genes in *Salvia miltiorrhiza* [13]. *PgERF120* modulates ginsenoside production in *Panax ginseng* [14], and Ii049 positively regulates lignan biosynthesis in *Isatis indigotica* [15]. These findings establish the AP2/ERF family as a functionally versatile transcription factor group with demonstrated roles in the regulation of medicinal compound biosynthesis.

Despite the established importance of AP2/ERF transcription factors in regulating plant secondary metabolism and the pharmacological significance of *S. chinensis*, a systematic genome-wide analysis of this gene family has not been reported in this species. Given that AP2/ERF members have been functionally linked to lignan and other specialized metabolic pathways in related species, we hypothesized that the AP2/ERF family in *S. chinensis* may include members that potentially regulate DBCOD biosynthesis, and that their tissue-specific expression patterns may correlate with the differential accumulation of lignans across organs. To test this hypothesis, we utilized the high-quality genome of *S. chinensis* [9] to conduct a comprehensive identification of AP2/ERF family members, discovering 144 *ScAP2/ERF* genes and categorizing them into five subfamilies. We systematically analyzed their phylogenetic relationships, structural features, chromosomal distribution, synteny, promoter cis-regulatory elements, and tissue-specific expression profiles. Our results provide a foundational framework for future functional studies aimed at elucidating the transcriptional regulatory mechanisms governing secondary metabolism in *S. chinensis*.

## Materials and methods

### Plant materials and treatment

During the fall of 2025, stems, leaves, fruits, and roots of *S. chinensis* were harvested from the Baicao Garden at Jilin Agricultural Science and Technology University. For each tissue type, three biological replicate samples were obtained. Each biological replicate consisted of pooled tissues from three individual plants to ensure representativeness. Right after collection, the samples were gently washed with deionized water to eliminate surface impurities, and any residual surface moisture was blotted away using filter paper. The tissues were then immediately frozen in liquid nitrogen and stored at −80 °C in an ultra-low temperature freezer until required.

*S. chinensis* seedlings at a height of 20–30 cm were selected for hormone treatment. A 1 mM methyl jasmonate (MeJA) solution was prepared and applied to the plants using two routes: 100 mL was sprayed onto the foliar surface, and another 100 mL was drenched into the soil around the roots. Leaf tissues were collected at 0, 6, 12, 24, and 48 h post-treatment. Immediately after harvesting, the leaves were rinsed thoroughly with distilled water, blotted dry, and flash‑frozen in liquid nitrogen. All samples were stored at −80°C until used for qRT‑PCR analysis. Three independent biological replicates were performed for each time point.

### Determination of schisandrol A content

The content of schisandrol A in different tissues of *S. chinensis* was determined using an Agilent LC 1260 Infinity II system equipped with a VWD detector. Separation was performed on an Origsil C18 column (5 μm, 4.6 × 150 mm) maintained at 30°C. The standard of schisandrol A.was purchased from Chengdu DeSiTe Biological Tech-nology Co. Ltd. (China). The mobile phase consisted of acetonitrile (A) and water (B) with the following gradient elution program: 0–10 min, 50% A; 10–15 min, 50–75% A; 15–25 min, 75% A; 25–30 min, 75–50% A. The flow rate was 1.0 mL/min, the injection volume was 10 μL, and the detection wavelength was set at 250 nm. The acquisition time was 30 min.

For sample preparation, roots, stems, leaves, and fruits were accurately weighed (to 0.0001 g) and extracted with 10 mL methanol. The mixtures were vortexed for 1 min, sonicated for 30 min, and then centrifuged at 12,000 rpm for 10 min. The supernatants were diluted 10-fold, filtered through a 0.22 μm membrane, and subjected to HPLC analysis. The standard curve for schisandrol A was constructed using the external standard method, with the regression equation y = 14.791192x − 4.549063 (R^2^ = 0.99998150), where y represents the peak area and x represents the concentration of schisandrol A.

### Identification of *ScAP2/ERF* family genes

To identify potential AP2/ERF family members in the S. chinensis genome, the HMMER (Hmmscan) toolkit was used to screen protein sequences for those containing the conserved AP2 domain (PF00847) with an E‑value threshold of 1e-5. Candidate proteins were further verified via BLASTp alignment to confirm the presence of an intact AP2 domain. Conserved domain structures were visualized using the “Visualize NCBI CDD Domain Pattern” function in TBtools. Basic physicochemical properties—including amino acid length, theoretical isoelectric point, and molecular weight—were computed with the ExPASy web platform [16]. Predictions for subcellular localization were made using the Wolf PSORT online tool.

### Categorization and evolutionary study of ScAP2/ERFs

Based on the classification and number of conserved domains in the ScAP2/ERF proteins, they are divided into the AP2 and RAV subfamilies [17]. The remaining ScAP2/ERF proteins, which possess only one AP2 domain, were also taken into consideration. To perform phylogenetic classification, all ScAP2/ERF proteins were combined with the AP2/ERF proteins from Arabidopsis thaliana to construct a phylogenetic tree. Maximum likelihood (ML) trees were generated using MEGA X software. An appropriate amino acid substitution model was first determined, and then the ML tree was constructed under this model, with bootstrap analysis conducted using 1,000 replicates and a partial deletion threshold of 80% [18]. The ultimate phylogenetic tree was displayed and labeled using the online tool iTOL (https://itol.embl.de/#) [19].

### Chromosomal mapping and sequence examination of *ScAP2/ERFs*

The chromosomal positions of the ScAP2/ERF genes, along with the overall length of each chromosome, were obtained from the *S. chinensis* genome. Gene structure visualization for the selected gene IDs was performed using the “Visualize Gene Structure (Basic)” tool within TBtools. To identify conserved motifs in the protein sequences encoded by the *ScAP2/ERF* genes, the online MEME suite (https://meme-suite.org/meme/tools/meme) was employed, with the number of motifs set to ten and the “Zero or One Occurrence Per Sequence (zoops)” model selected for motif distribution [20]. The resulting conserved motifs were visualized using the “Visualize MEME/MSAT Motif Pattern” function in TBtools. The structural organization of *ScAP2/ERF* genes—including untranslated regions (UTRs) and exons—was extracted from the *S. chinensis* genome annotation and subsequently displayed using TBtools. Promoter regions were defined as the 2 kb sequences immediately upstream of the translation start site for each *ScAP2/ERF* gene, and cis-acting elements within these promoters were then identified using the PlantCARE database.

The arrangement of *ScAP2/ERF* genes on chromosomes was displayed using the ‘Gene Location Visualize from GTF/GFF’ feature in TBtools. To investigate syntenic relationships, MCScanX software was employed for both intragenomic synteny analysis among *ScAP2/ERF* genes within the *S. chinensis* genome and intergenomic synteny analysis between *S. chinensis* and *A. thaliana* genomes. The resulting synteny maps, showing both within-genome and cross-genome comparisons, were displayed using Circos software.

### Profiling the expression of *ScAP2/ERFs* in different tissue types

Transcriptome data for various *S. chinensis* tissues (roots, stems, leaves, and fruits) were retrieved from the Beijing Institute of Genomics (Chinese Academy of Sciences) Big Data Center (accession: CRA019045 / PRJCA030150) [9]. Corresponding FPKM values were then extracted, and a heatmap of ScAP2/ERF family expression profiles was created using the “HeatMap” tool in TBtools.

Total RNA extracted from plant tissues using a commercial kit (Takara, Japan) was reverse-transcribed into cDNA with the PrimeScript™ RT reagent kit (Takara). Using Primer Premier 6 (Premier, Canada), gene-specific primers were designed for qRT-PCR, with GAPDH as the internal control (S1 Table in S1 File). Primer amplification efficiency was verified for each primer pair using a five-point standard curve (R^2^ > 0.99), yielding efficiencies between 90% and 110%. qRT-PCR was carried out on a QuantStudio 5 system (Applied Biosystems, USA). Each 20 µL reaction included 10 µL of 2 × ChamQ Universal SYBR qPCR Master Mix, 0.5 µL each of forward and reverse primers, 1 µL of cDNA template, and 8 µL of ddH_2_O. The thermal profile was: 95 °C for 5 min (initial denaturation), then 40 cycles of 95 °C for 10 s, 60 °C for 30 s, and 72 °C for 30 s, followed by a melting curve step (95 °C for 15 s, 60 °C for 1 min, 95 °C for 1 s with continuous fluorescence acquisition). No-template controls (NTC) were included for each primer pair to rule out contamination. All reactions were performed in three technical replicates for each of the three biological replicates, and relative expression levels were determined using the 2^-ΔΔCt^ method.

Statistical analysis was performed using one-way analysis of variance (ANOVA) followed by Tukey’s honestly significant difference (HSD) test for pairwise comparisons, implemented in SPSS software (Version 26.0; IBM Corp., Armonk, NY, USA). A P-value < 0.05 was considered statistically significant. All figures displaying expression data represent mean values ± standard error (SE).

## Results

### Identification and protein physicochemical properties of ScAP2/ERF in *S. chinensis*

Members containing the AP2 domain were screened using HMMER (Pfam accession PF00847), and further filtering based on CDD analysis ultimately identified 144 protein sequences possessing complete AP2/ERF domains (S2 Table in S1 File). Physicochemical characterization revealed that the amino acid lengths of the ScAP2/ERF proteins ranged from 112 (ScERF103) to 781 (ScERF136), with the AP2 subfamily proteins generally exhibiting larger sizes (average 445.90 aa). The isoelectric points ranged from 4.53 to 9.80, with most DREB and ERF-B3 subfamily members showing basic characteristics. All proteins exhibited negative GRAVY values (−0.981 to −0.222), indicating their hydrophilic nature, which is consistent with their roles as transcription factors that function in aqueous nuclear environments to bind DNA and regulate gene expression. The instability index ranged from 33.55 to 80.39; proteins with instability indices above 40 are classified as unstable, suggesting that a subset of ScAP2/ERF proteins may undergo rapid turnover, potentially facilitating dynamic transcriptional responses to developmental or environmental cues. Subcellular localization prediction showed that 80% of the proteins were localized in the nucleus, consistent with their function as transcription factors.

### Phylogenetic analysis of the *ScAP2/ERF* genes

Using 140 AtAP2/ERF proteins from *A. thaliana* and 144 ScAP2/ERF proteins from *S. chinensis*, a phylogenetic tree was developed. Following the classification guidelines for the AP2/ERF family in *A. thaliana*, the ScAP2/ERF members were sorted into five subfamilies: ERF, DREB, AP2, RAV, and Soloist, consisting of 62, 54, 21, 6, and 1 members, respectively (Fig 1). Among these, the DREB subfamily was further subdivided into five groups (A1, A2/A3, A4, A5, and A6) with 13, 8, 14, 15, and 5 members, respectively; within the ERF subfamily, there are six groups: B1, B2, B3, B4, B5, and B6, with 13, 8, 29, 3, 3, and 6 members in each group, respectively. Alternatively, as per the classification system proposed by Nakano and colleagues [21], the DREB subfamily is categorized into Groups I through IV, while the ERF subfamily is divided into Groups V through X. Specifically, Group A6 corresponds to Group I, Group A5 to Group II, Groups A1 and A4 belong to Group III, and Group A2/A3 belongs to Group IV; Group B1 corresponds to Group VIII, Group B2 to Group VII, Group B3 to Group IX, Group B4 to Group X, and Group B5 to Group VI; while Groups V, Xb‑L, and VI‑L are all assigned to Group B6. Notably, the further subdivisions VI‑L and Xb‑L, which are present in the AtAP2/ERF family, were not observed in *S. chinensis*.

**Fig 1:**
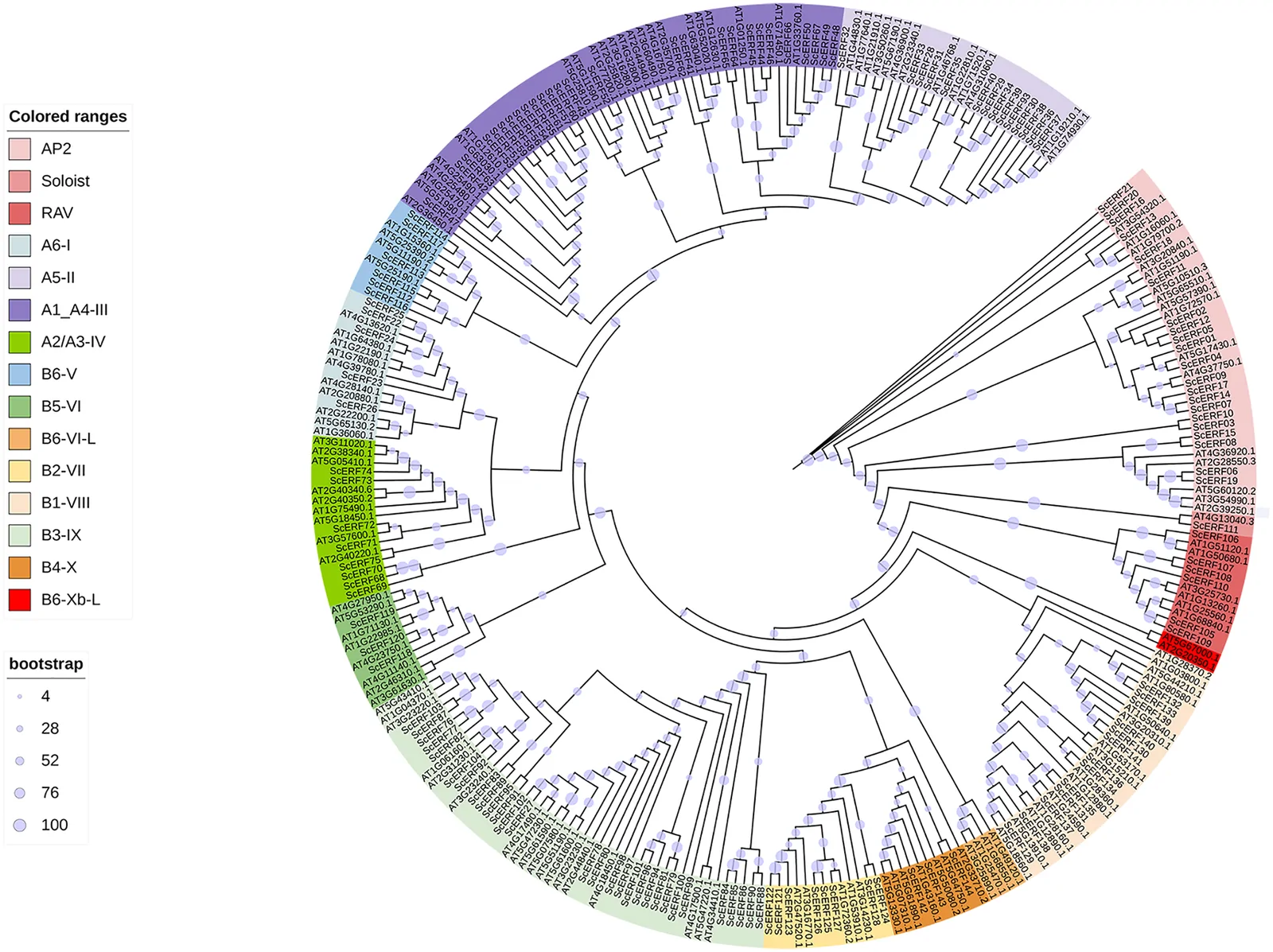
Phylogenetic tree for the ScAP2/ERFs and AtAP2/ERFs. The tree includes 144 ScAP2/ERF proteins from *S. chinensis* (labeled with ‘Sc’ prefix) and 140 AtAP2/ERF proteins from *A. thaliana* (labeled with ‘At’ prefix). Proteins are color-coded by subfamily: ERF (green), DREB (blue), AP2 (red), RAV (orange), and Soloist (purple).

### Investigation into the conserved domains and motifs of ScAP2/ERF

The alignment revealed that all ScAP2/ERF proteins contain the AP2 domain, consistent with the CDD search results showing an AP2 domain in each sequence (Fig 2). Among them, AP2 subfamily members harbor two repeated AP2 domains, RAV subfamily members possess both AP2 and B3 domains, whereas members of other subfamilies contain only one AP2 domain. In addition, some proteins contain additional domains such as the FHY3 superfamily. Conserved motif analysis of the 144 ScAP2/ERF proteins using MEME identified ten motifs, designated motif1 to motif10 (Fig 3). Motif1 is essential for the AP2/ERF family and was present in every sequence. Most AP2 subfamily members contain motif1, motif4, motif10, and two copies of motif3. All DREB subfamily members contain motif1 and motif3; additionally, members of the A1 and A4 groups possess motif2, motif5, motif7, motif8, and motif9, A2/A3 group members contain motif2, and A5 group members harbor motif6 and motif7. Group A6 is similar to A2/A3, containing motif1, motif2, and motif3, and these groups cluster into a large branch on the phylogenetic tree. ERF subfamily members, divided into six groups (B1–B6), resemble the DREB subfamily in containing motif1 and motif3. Within the B1 group, all members except ScERF131 and ScERF137 contain motif6, motif1, and motif3. All B2 group members contain motif2, motif1, and motif3. In the B3 group, with the exception of ScERF79, all members (as well as those of the B4 group) contain motif2, motif1, and motif3. All RAV subfamily members contain motif1 and motif3, while the Soloist subfamily member contains only motif1.

**Fig 2:**
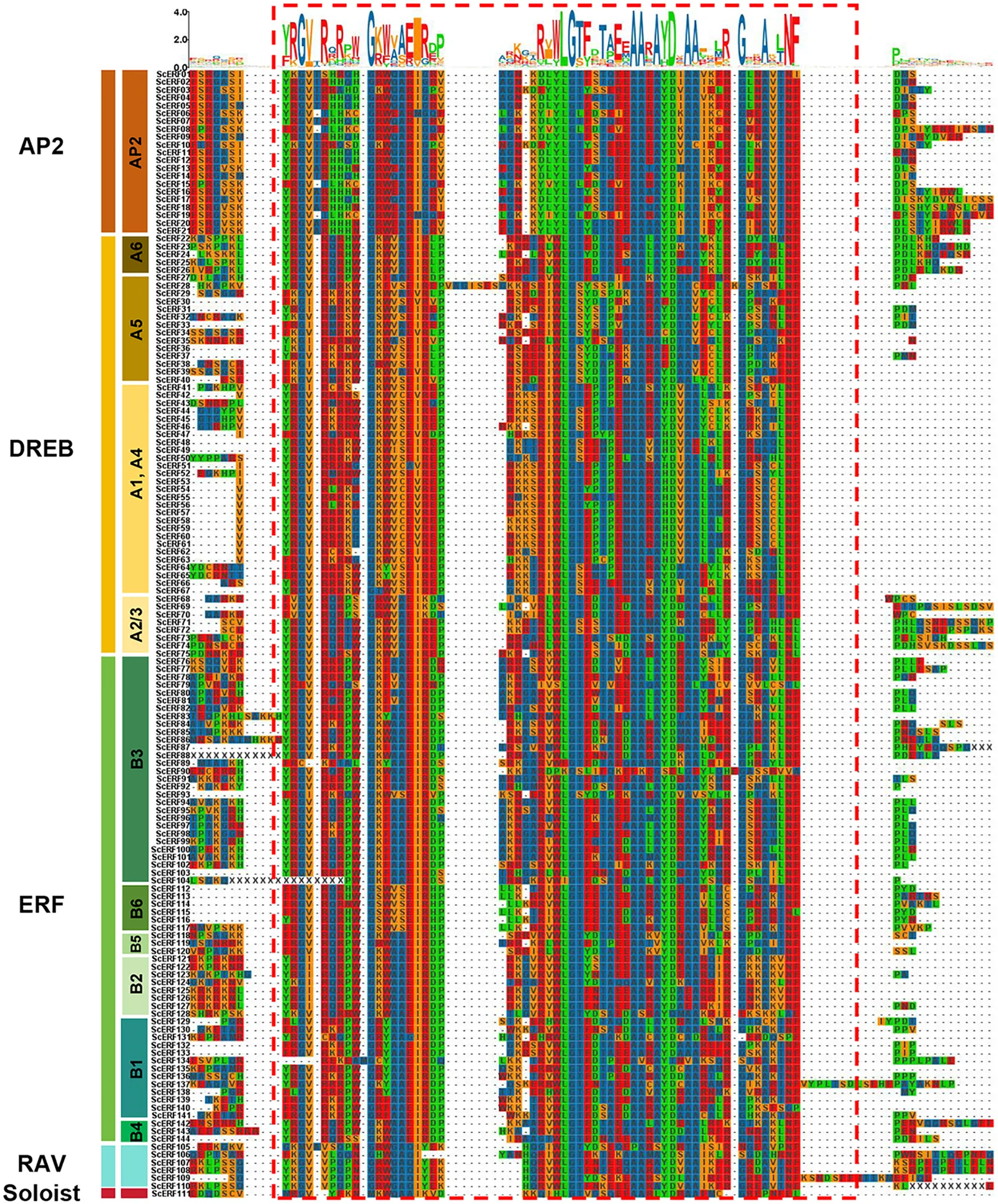
Multiple sequence alignment of ScAP2/ERFs. AP2 domain are displayed as red box. Rectangles of different colors represent different cis components.

**Fig 3:**
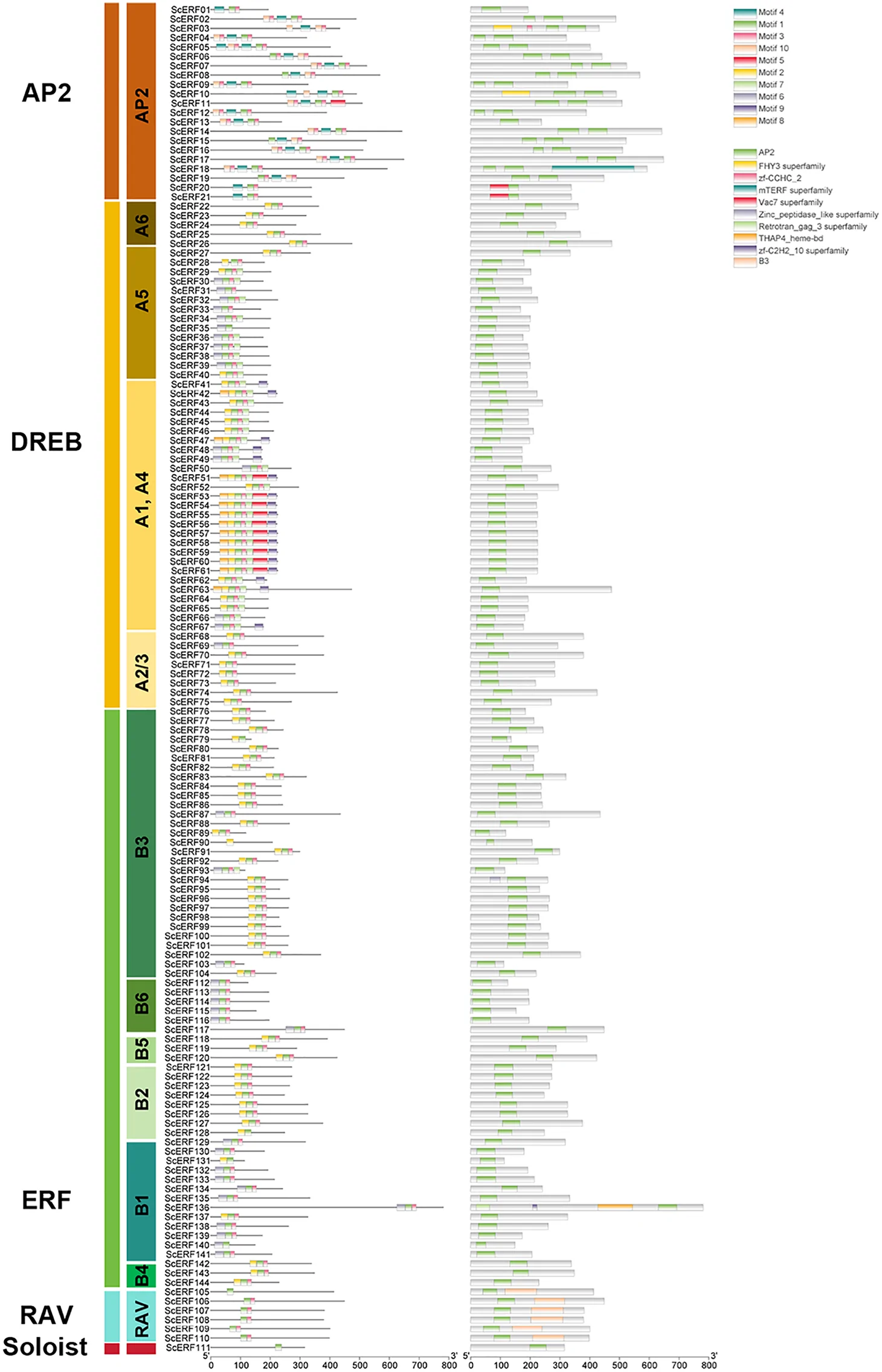
The structural domains and preserved patterns of ScAP2/ERFs. Ten conserved motifs were identified using MEME and are displayed as colored boxes. The AP2 domain (motif 1) is present in all proteins. AP2 subfamily members contain two AP2 domains, while RAV members contain both AP2 (motif 1) and B3 (motif 2) domains.

### Chromosomal mapping and colinearity analysis of *ScAP2/ERFs*

The 144 *ScAP2/ERF* genes were found to be unevenly located on the 14 chromosomes according to chromosomal localization analysis. Among them, chromosomes Chr1, Chr2, and Chr11 harbored the largest numbers of genes, with 22, 20, and 15 genes, respectively, whereas chromosome Chr4 contained the fewest, with only four genes (Fig 4). Clustered distribution patterns were observed on chromosomes Chr1, Chr2, Chr6, Chr10, Chr11, and Chr13, while the genes were relatively dispersed on other chromosomes. To determine whether these clustered arrangements result from genuine duplication events, we performed pairwise Ka/Ks-based evolutionary analysis on all 40 adjacent gene pairs within the tandem arrays (S3 Table in S1 File). The results confirmed that these clusters indeed originated from tandem duplications, with divergence times ranging from recent (e.g., Chr8: ScERF84/85, Ks = 0.057; Chr10: ScERF64/65, Ks = 0.039) to ancient (pS ≥ 0.75, Ks saturation), and even including ultra-recent pairs with zero synonymous substitutions (Chr10: ScERF20/21; Chr11: ScERF125/126). Notably, the consistently low Ka/Ks values across most evaluable pairs indicate that these duplicated copies have been predominantly maintained by purifying selection. Genes located in these clusters may share similar functions or participate in the same biological processes, and their tandem-duplicated nature provides a genomic basis for functional redundancy or subfunctionalization within the ScAP2/ERF family.

**Fig 4:**
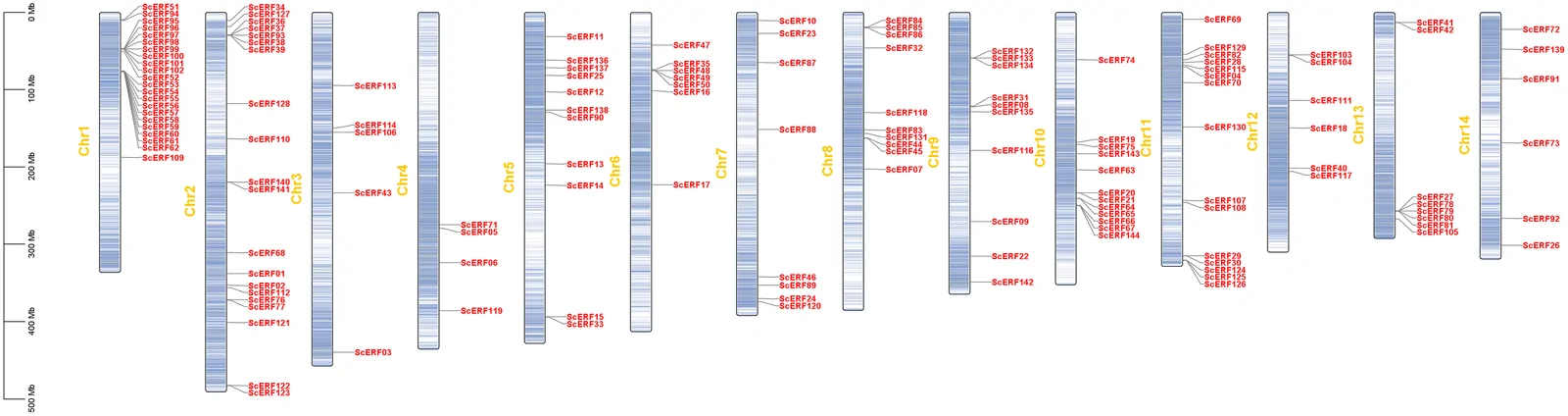
Chromosomal distribution of the ScAP2/ERFs.

Synteny analysis within the *S. chinensis* genome was performed using MCScanX. The results revealed 26 pairs of *ScAP2/ERF* genes distributed across 14 chromosomes, mainly located on Chr2, Chr5, Chr6, Chr9, Chr10, and Chr11 (Fig 5A). These gene pairs are likely derived from gene duplication events and may therefore share similar biological functions. To evaluate the selection pressure on these retained duplicates, we calculated the Ka/Ks ratios for all 26 pairs (S4 Table in S1 File). Except for one saturated pair (ScERF100/ScERF27, pS ≥ 0.75), the remaining 25 pairs exhibited Ka/Ks values significantly below 1, ranging from 0.156 to 0.490, indicating that these syntenic duplicates are predominantly under purifying (negative) selection rather than positive selection. This suggests strong functional constraints and dosage balance maintenance after duplication. Gene duplication generally promotes mutations that can lead to novel functions or generate genes essential for plant adaptation to new environments. Furthermore, comparative synteny analysis between the *S. chinensis* genome and that of the model plant *A. thaliana* identified 39 *ScAP2/ERF* genes exhibiting syntenic relationships with their counterparts in *A. thaliana* (Fig 5B). This finding is of great significance for elucidating the evolutionary history, functional conservation, and functional diversity of the *ScAP2/ERF* gene family.

**Fig 5:**
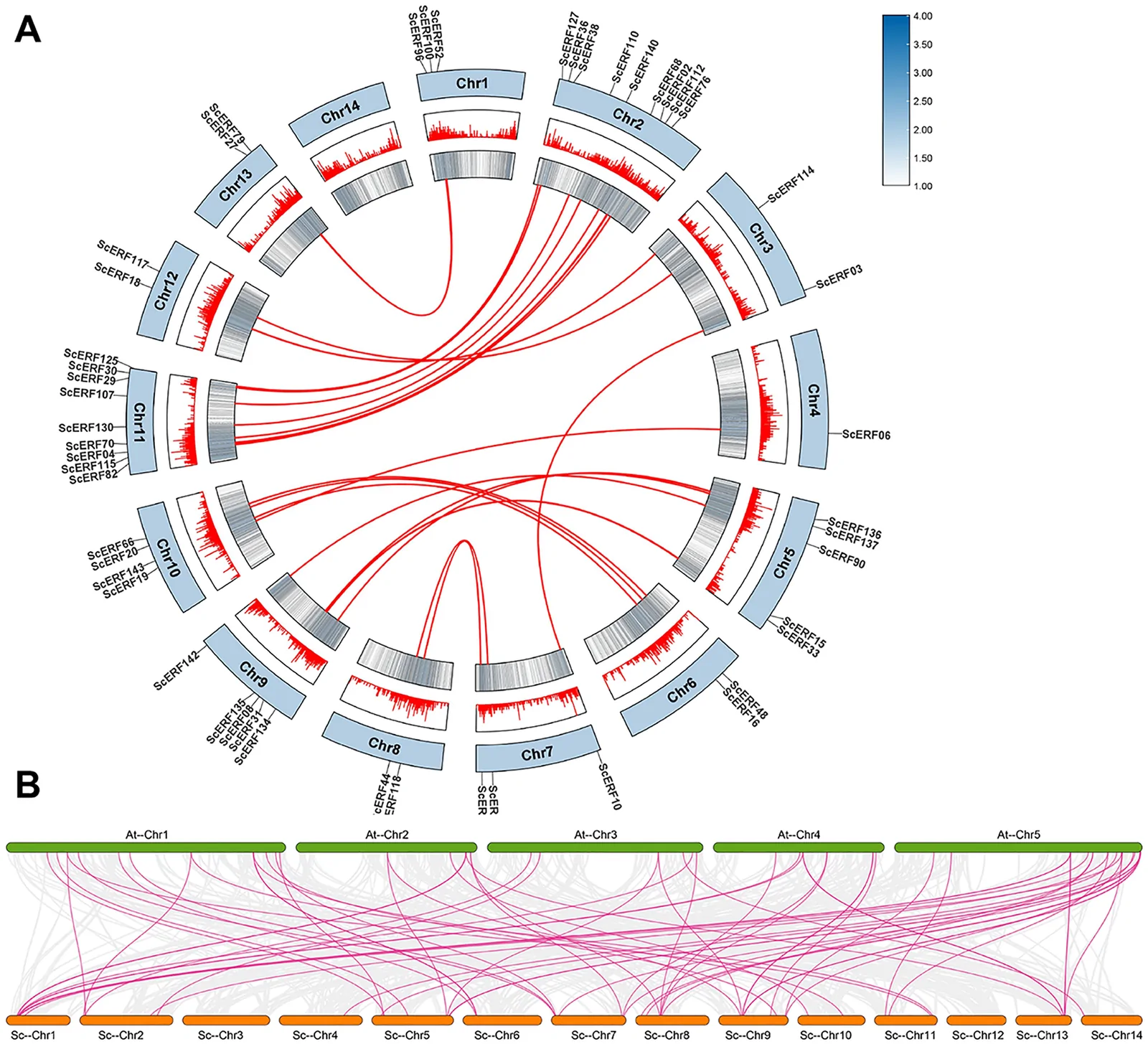
Analysis of collinearity among and between species of ScAP2/ERFs. A. Analysis of collinearity among ScAP2/ERF family genes, with homologous genes connected by red lines. B. Examination of collinearity between AP2/ERFs in *S. chinensis* and *A. thaliana.*

### Evaluation of cis-acting factors in the promoters of *ScAP2/ERFs*

The PlantCARE online tool facilitated the extraction and analysis of 2000 bp promoter sequences upstream of the start codon (ATG) to identify cis-acting elements. A total of 25 types of regulatory elements were identified, which were categorized into three major functional groups: hormone-responsive elements, stress-responsive elements, and growth/development-related elements. Among them, light-responsive elements, MeJA-responsive elements, ABA-responsive elements, and anaerobic induction elements were the most abundant, accounting for 37%, 14%, 13%, and 10% of the total, respectively. This suggests that light signaling, MeJA, ABA, and anaerobic conditions may play key roles in regulating the expression of *ScAP2/ERF* genes (Fig 6). Light-responsive elements were the most numerous, with a total count of 1475, indicating that light conditions exert the most significant influence on *ScAP2/ERFs* expression during growth and development. Other elements identified included auxin-responsive, gibberellin-responsive, salicylic acid-responsive, low-temperature-responsive, and wound-responsive elements, as well as meristem- and seed-specific expression elements, implying that the expression regulation of *ScAP2/ERF* genes is a complex process involving multiple environmental factors and hormonal signals. The distribution and composition of these cis-acting elements provide important clues for further functional and regulatory studies of the *ScAP2/ERF* gene family in *S. chinensis.*

**Fig 6:**
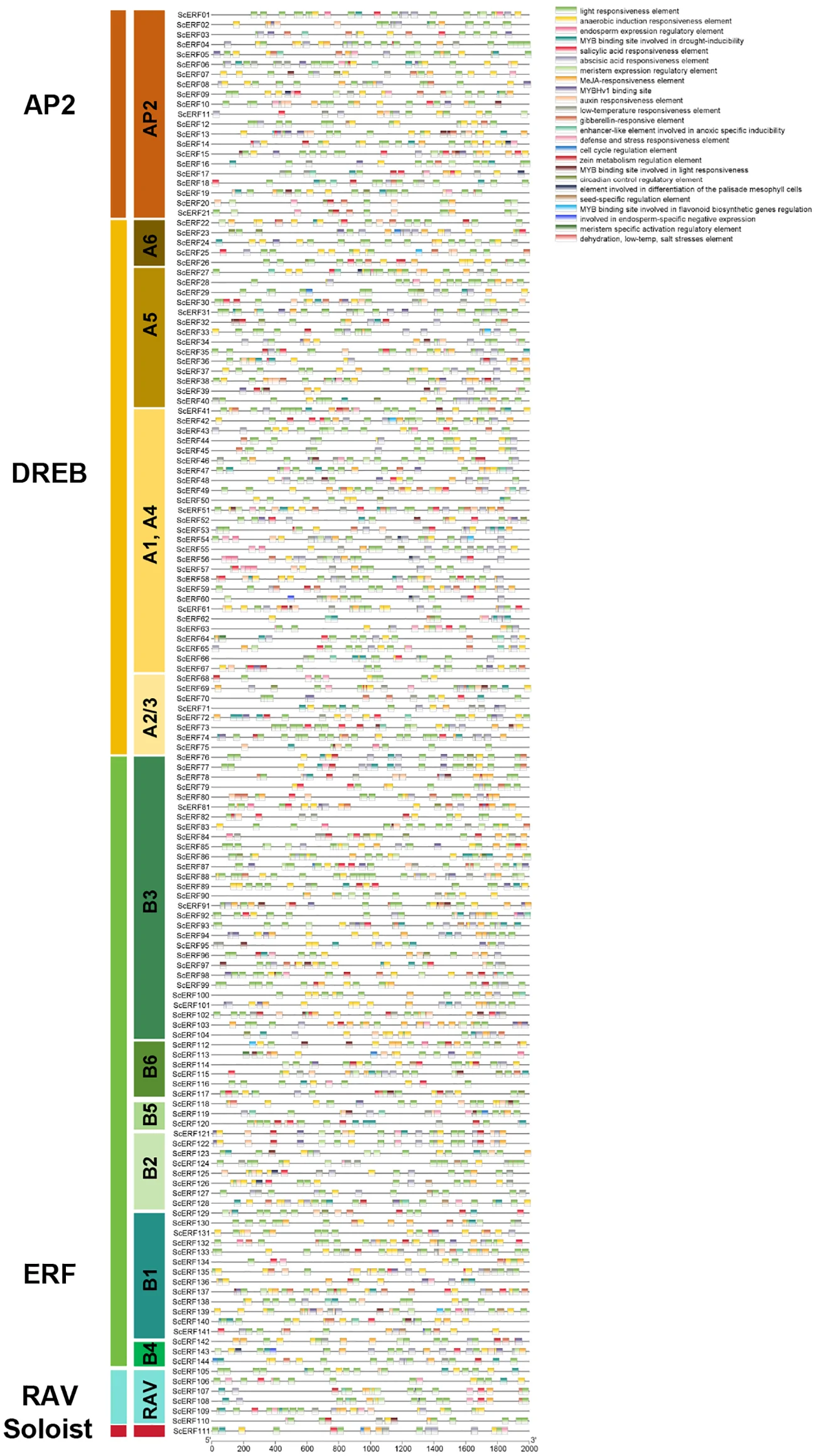
Investigation into the cis-regulatory components of *ScAP2/ERFs.* Rectangles of different colors represent different cis components.

### Expression profiles of *ScAP2/ERFs* from different tissues

The 144 *ScAP2/ERF* members exhibited diverse expression patterns across roots, stems, leaves, and fruits of *S. chinensis*, with 122 genes detectably expressed in at least one tissue (Fig 7, S5 Table in S1 File). Specifically, the numbers of highly expressed genes in leaves, stems, roots, and fruits were 29, 24, 36, and 33, respectively. Notably, ten *ScAP2/ERF* genes (including *ScERF100, ScERF107, ScERF40, ScERF38, ScERF05, ScERF94, ScERF104, ScERF108, ScERF102*, and *ScERF64*) showed fruit‑specific high expression (Fig 7B). Previous studies have demonstrated that lignans in S. chinensis are primarily enriched in fruits, with distinct compound-specific distribution patterns among tissues, and the dried mature fruit is the designated medicinal part according to the Chinese Pharmacopoeia (2025 edition) [9]. Schisandrol A is considered a quality marker component, with its content across different parts of *S. chinensis* showing a trend of fruits > stems > roots > leaves (Fig 8A-8B). The qRT-PCR validation of the ten fruit-preferential *ScAP2/ERF* genes showed expression patterns consistent with the RNA-seq data. In fruits, *ScERF94*, *ScERF100*, *ScERF102*, and *ScERF104* exhibited markedly higher transcript levels than in other tissues; *ScERF05* and *ScERF64* showed higher expression in stems and roots, while *ScERF107* showed the lowest expression in roots (Fig 8C). Furthermore, given the presence of numerous MeJA-responsive cis-elements in the promoters of ScERFs, we treated S. chinensis leaves with MeJA and subsequently examined the expression of the ten *ScAP2/ERF* genes. The results showed that *ScERF38*, *ScERF100*, *ScERF104*, *ScERF107*, and *ScERF108* rapidly peaked at 6 h post-MeJA induction before declining quickly. *ScERF05*, *ScERF40*, and *ScERF64* peaked at 12 h post-induction followed by decreased expression, whereas *ScERF102* exhibited a decrease at 6 h, peaked at 12 h, and then declined (Fig 8D). The regulatory mechanism by which *ScERF* influences the lignan biosynthesis in *S. chinensis* requires further investigation.

**Fig 7:**
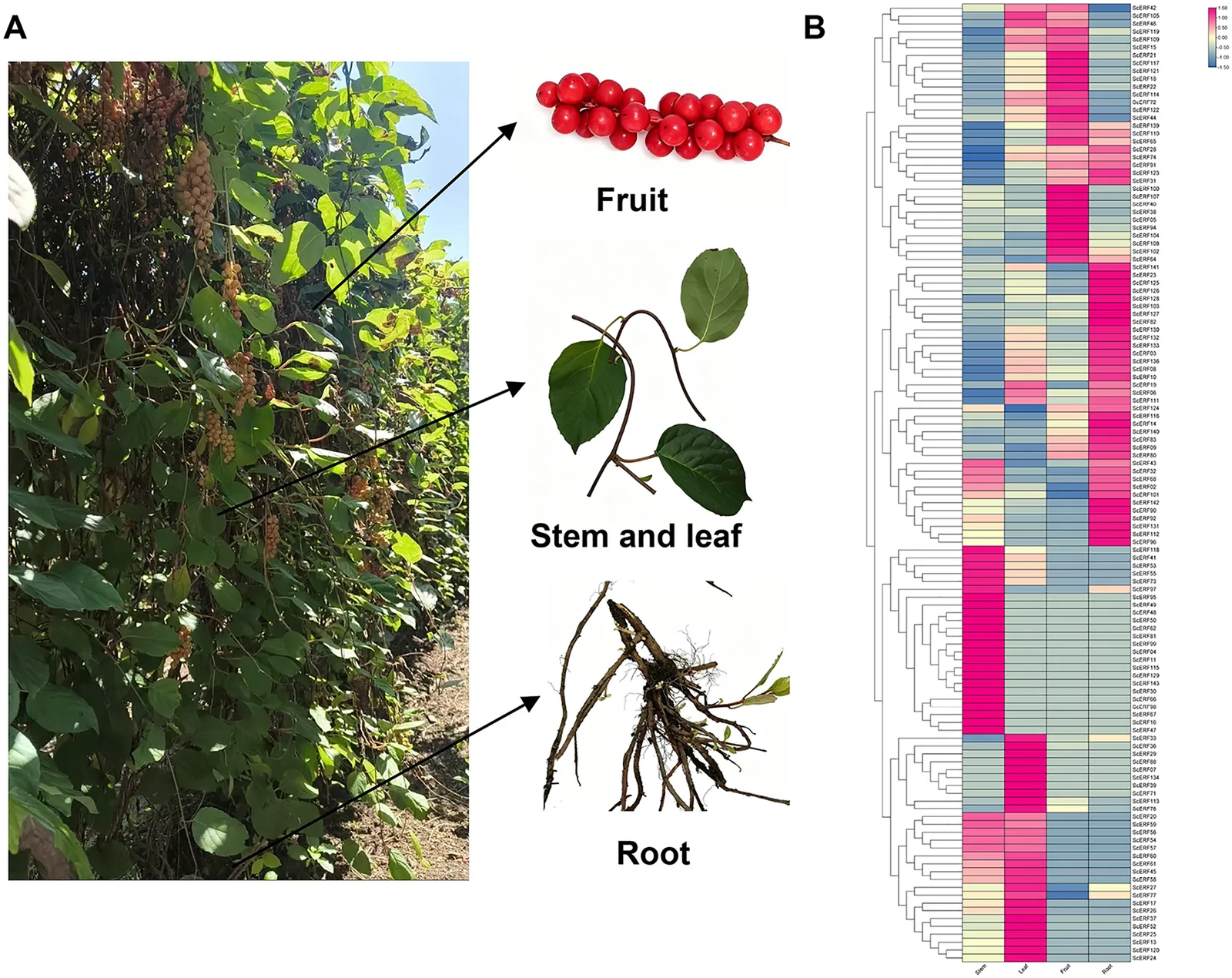
Distribution of *ScAP2/ERFs* expression across various *S. chinensis* tissues. A. The morphology of S. chinensis leaf, stem, fruit, and root. B. FPKM changes of *ScAP2/ERFs* at different tissues.

**Fig 8:**
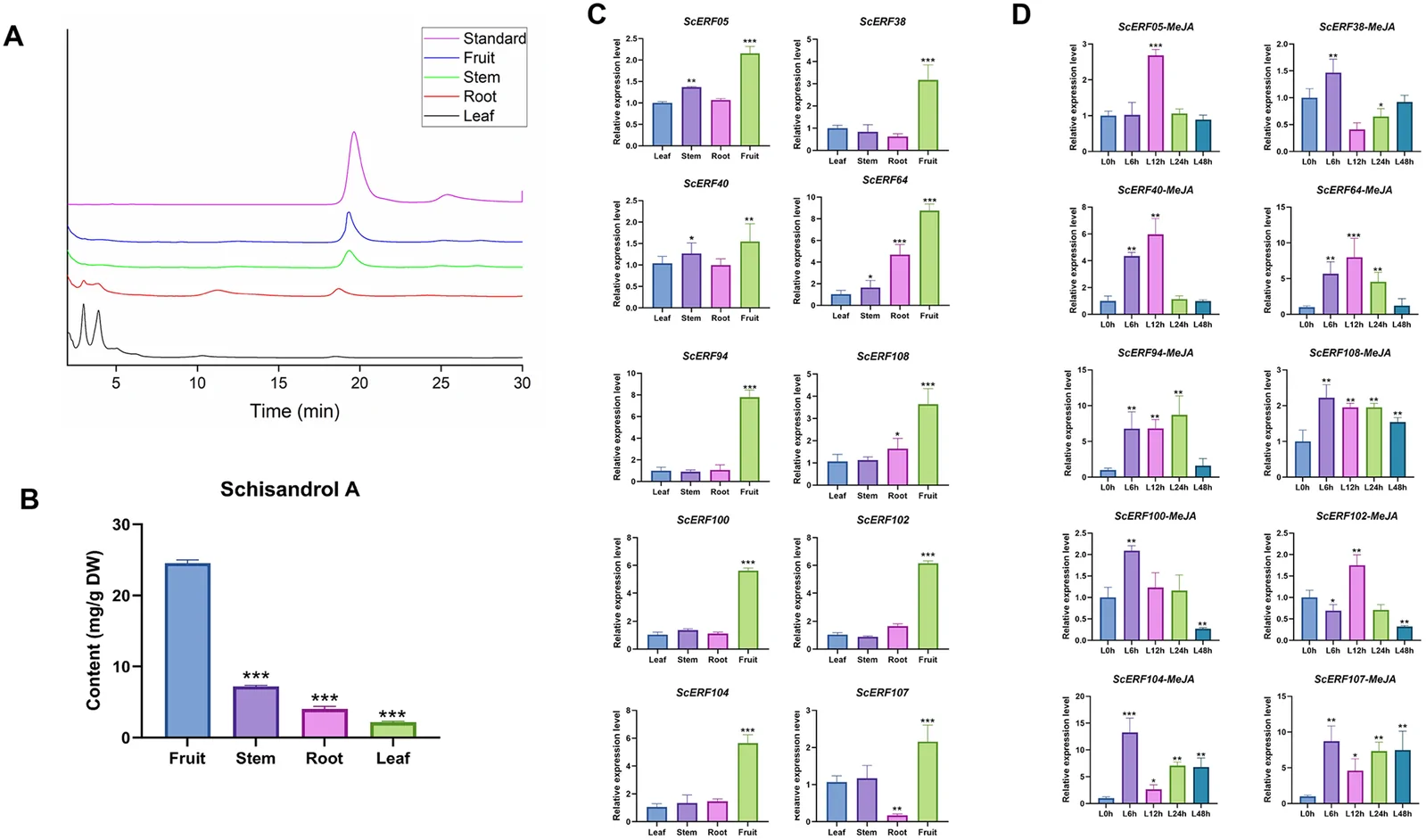
The contents of Schisandrol A in different tissue and exprssion of key *ScAP2/ERFs.* A. HPLC chromatogram of Schisandrol A in different tissue; B. The contents of Schisandrol A in different tissue; C. The qRT-PCR analysis of 10 fruit-specificially highly expressed *ScAP2/ERFs*. D. The qRT-PCR analysis of 10 fruit-specificially highly expressed ScAP2/ERFs following MEJA induction. Data are presented as mean ± SD of three biological replicates. * indicates *p* < 0.05, ** indicates *p* < 0.01, *** indicates *p* < 0.001.

## Discussion

The AP2/ERF transcription factor family constitutes a major group of transcriptional regulators in plants. In this study, we identified 144 ScAP2/ERF members from the chromosome-level genome of *S. chinensis*, categorized into five subfamilies. This number is comparable to that reported for other species This number is comparable to that reported for other species, such as *S. miltiorrhiza* (169), *Oryza sativa* (164), and *Eschscholzia californica* (134) [22–24]. The predominance of the ERF and DREB subfamilies (accounting for over 80% of the total) is consistent with observations in most land plants, including *Capsicum annuum* [25], in *Nicotiana tabacum* [26], and in *Coptis chinensis*, 43 ERF and 32 DREB members have been identified [27], reflecting the crucial roles of these subfamilies in stress responses.

Conserved motif analysis revealed that the universal presence of the AP2 core domain (motif 1) is a hallmark of this family, with AP2 subfamily members containing two AP2 domains and RAV members harboring an additional B3 domain. The additional motifs enriched in specific subfamilies (e.g., motifs 2, 5, 7, 8, and 9 in groups A1/A4) may contribute to modulating DNA-binding specificity or transcriptional activation functions, as has been suggested for ERF factors in *E. californica* [22]. Regarding chromosomal distribution and duplication mechanisms, *ScAP2/ERF* genes exhibit clustered distribution patterns on chromosomes Chr1, Chr2, and Chr11, which is consistent with findings in *Boehmeria nivea*, where at least 30% of *AP2/ERF* genes are arranged in clusters [28], as well as with observations in rose that gene clusters are significantly associated with tandem duplication events [29]. Synteny analysis identified 26 pairs of intra‑genomic duplication events and 39 orthologous gene pairs between *S. chinensis* and *A. thaliana,* suggesting that segmental and tandem duplication are the primary mechanisms driving driving AP2/ERF family expansion, similar to findings in other medicinal plants [13,14].

Analysis of promoter cis-acting elements revealed that light-responsive elements comprised the highest percentage (37%), followed by MeJA-responsive (14%), ABA-responsive (13%), and anaerobic induction elements (10%). The prevalence of light-, MeJA-, and ABA-responsive elements indicates that *ScAP2/ERF* genes are integral to light signaling and phytohormone-mediated secondary metabolism. Notably, AP2/ERF transcription factors are well-documented downstream effectors of the JA signaling pathway, which serves as a central hub for plant secondary metabolism, suggesting that these *ScAP2/ERF* genes may participate in lignan biosynthesis by transducing hormonal signals to downstream target genes [30]. For instance, in *I. indigotica*, MeJA treatment significantly induced multiple *IiAP2/ERF* genes co-expressed with lignan biosynthetic enzymes [24], This hypothesis is reinforced by recent advances in MeJA-elicited lignan production and the enzymatic machinery of *S. chinensis.* Transcriptome analysis of MeJA-treated *S. chinensis* identified key O-methyltransferase (OMT) genes that catalyze the methylation steps essential for DBCOD lignan structural diversification [31]. Similarly, MeJA elicitation in *S. chinensis* microshoot cultures significantly enhanced total lignan accumulation, confirming MeJA as an effective elicitor for activating the lignan biosynthetic machinery [32]. In accordance with these findings, our promoter analysis revealed that MeJA-responsive elements account for 14% of the cis-acting elements in *ScAP2/ERF* promoters, and ten fruit-preferential *ScAP2/ERF* genes exhibited rapid and transient induction upon MeJA treatment, with peaks at either 6 h or 12 h. Given that AP2/ERFs act as downstream effectors of JA signaling, these transcription factors likely mediate MeJA-induced lignan biosynthesis by regulating downstream structural genes, including OMTs. The fruit-preferential expression of these *ScAP2/ERF* genes, together with the fruit-dominant accumulation of schisandrol A (fruits > stems > roots > leaves), further suggests that this regulatory module is particularly active in the medicinal fruit of *S. chinensis*.

Expression analysis provided important clues regarding the potential roles of ScAP2/ERF transcription factors in tissue-specific processes. Ten genes exhibited fruit-specific high expression, which was validated by qRT-PCR. The dried mature fruit of *S. chinensis* is the designated medicinal part in the *Chinese Pharmacopoeia* (2025) and and serves as the primary organ for DBCOD lignan accumulation [33,34]. Our determination of schisandrol A content in different tissues also suggested this. The spatial coincidence between fruit-specific gene expression and lignan enrichment is consistent with a potential regulatory role, although direct causal relationships remain to be established [35]. In *I. indigotica*, Ii049 promotes lariciresinol accumulation by directly activating the promoters of *PAL* and *CCR* [15]; in loquat, EjERF39 activates *Ej4CL1* transcription to promote lignin accumulation [36]. By analogy, the fruit‑specific highly expressed *AP2/ERF* genes in *S. chinensis* may play analogous roles by potentially recognizing GCC-box, CE1, or DRE elements in the promoters of structural genes such as PAL, C4H, 4CL, CCR, and CAD. The precise regulatory modes require further experimental validation.

## Limitations

Several limitations of this study should be acknowledged. Confirming these predicted regulatory interactions will require experimental approaches such as yeast one-hybrid, dual-luciferase reporter, or transient expression assays. Several limitations should be acknowledged. This study primarily involved genome-wide identification of the *AP2/ERF* gene family in *S. chinensis* and expression analysis of key *AP2/ERF* genes; functional validation was not performed. Consequently, the proposed regulatory roles of *ScAP2/ERF* genes in secondary metabolism remain correlative and require experimental confirmation through approaches such as hairy root transformation, promoter activity assays, or hormone treatment analyses.

## Conclusion

In conclusion, this study provides a comprehensive genome-wide analysis of the AP2/ERF transcription factor family in *S. chinensis*. A total of 144 *ScAP2/ERF* genes were identified and phylogenetically classified into five subfamilies. Conserved motif and domain analyses confirmed the characteristic AP2 domain architecture across all family members. Chromosomal localization revealed uneven distribution with distinct gene clusters, while promoter analysis identified multiple stress- and hormone-responsive cis-elements, with MeJA-responsive elements accounting for 14%. Synteny analysis uncovered both segmental duplications within the *S. chinensis* genome and orthologous relationships with *A. thaliana*. Ka/Ks analysis of duplicated gene pairs indicated predominant purifying selection. Notably, ten genes—*ScERF100, ScERF107, ScERF40, ScERF38, ScERF05, ScERF94, ScERF104, ScERF108, ScERF102*, and *ScERF64*—exhibited fruit-preferential expression and were rapidly induced by MeJA, with expression peaks at either 6 h or 12 h. Given that schisandrol A, the quality marker of *S. chinensis*, accumulates predominantly in fruits (fruits > stems > roots > leaves), these fruit-preferential and MeJA-responsive *ScAP2/ERF* genes are likely involved in regulating lignan biosynthesis. While the present study is limited by its computational and descriptive nature, these findings establish a fundamental resource for future experimental investigation of the evolutionary history and functional diversification of the AP2/ERF family in *S. chinensis* and provide candidate genes for experimental functional validation.

## Supporting information

S1 File S1 Table. The sequences of primers used in qRT-PCR. **S2 Table.** Physicochemical properties of ScAP2/ERFs. **S3 Table.** Ka/Ks calculation of 26 gene pairs. **S4 Table.** Tandem repeat gene pair for KaKs calculation. **S5 Table.** The gene expression of ScAP2ERF.
(RAR)
