# Supplementary material for: Comprehensive Analysis of the AP2/ERF Transcription Factor Family in Schisandra chinensis and Its Implications for secondary metabolism
Source: PLoS One. 2026 Oct 15;21(10):e0361017. doi: 10.1371/journal.pone.0361017 (PMC13671533; doi:10.1371/journal.pone.0361017)
Supplement: Supplementary file 1 [file pone.0361017.s002.docx]

The manuscript presents a comprehensive study involving genome-wide identification, characterization, subcellular localization, phylogenetic analysis, and other aspects of the AP2/ERF gene family in *Schisandra chinensis* along with the tissue specific expression profiling. However, the manuscript lacks a clear rationale and explanation of the study's importance.
In conclusion, I recommend resubmitting the manuscript for further consideration. Prior to resubmission, the identified issues and recommended improvements are outlined below:

The inclusion of "lignan biosynthesis" in the title is not adequately supported by the data presented in the manuscript. No comprehensive analysis or functional investigation of lignan biosynthesis is provided throughout the study. The authors should revise the title to accurately reflect the actual scope and findings of the work.

**Introduction-**

- The introduction lacks a coherent scientific storyline. The authors should reorganize the introduction to build a stronger rationale leading to the central objective of the study.
- A substantial portion of the introduction is devoted to background information that does not directly contribute to the research objective. This results in an unnecessarily lengthy introduction and distracts the reader from the main scientific question.
- The introduction fails to adequately justify why AP2/ERF transcription factors was chosen for investigation. A stronger mechanistic rationale supported by previous studies is needed.

**Materials and Methods-**

- The formatting of botanical names is inconsistent throughout the manuscript. All scientific names of plant species should be written in italics. in accordance with standard taxonomic conventions. Please check and correct this throughout the manuscript.
- Please clarify the rationale for constructing the phylogenetic tree using only *Arabidopsis thaliana* and the study species. A phylogenetic tree based on only two species provides limited evolutionary insight and is generally insufficient to support evolutionary inferences. Inclusion of homologs from additional representative species would substantially strengthen the phylogenetic analysis.
- The cis-regulatory element analysis appears to rely on conventional prediction resources. The authors are encouraged to explore and compare results with more recent and comprehensive plant cis-regulatory databases or motif resources to improve the reliability and biological relevance of the predicted regulatory elements.
- Please justify the rationale for using *Arabidopsis thaliana* as the reference species for the synteny analysis.
- The authors have analyzed the tissue-specific expression pattern of AP2/ERF genes; however, it is unclear how these expression profiles were associated with lignan biosynthesis. Were lignan contents or compositions quantified in the corresponding tissues?
- AP2/ERF transcription factors constitute a large and functionally diverse family involved in numerous biological processes, including plant growth, development, and responses to various biotic and abiotic stresses. Therefore, tissue-specific expression alone is insufficient to infer their specific role in lignan biosynthesis.
- The qRT-PCR validation appears to be based only on biological replicates. Please clarify whether technical replicates were performed.
- The manuscript does not specify the statistical methods used to analyze the qRT-PCR data. Please clearly describe the statistical software, tests employed and significance thresholds used for the analysis.

**Results-**

- The subcellular localization of the candidate protein(s) has been predicted only through computational approaches. Experimental validation, such as transient expression of GFP fusion constructs in *Nicotiana benthamiana* leaves, would substantially strengthen the results.
- Figure 2 is difficult to interpret as the multiple sequence alignment is not organized according to the AP2/ERF subfamilies identified in the phylogenetic tree. To improve clarity authors should rearrange the alignment by grouping sequences according to their respective subfamilies.
- Tissue-specific expression analysis is a key component of this study; however, no representative images of the plant tissues used for qRT-PCR are provided. The authors are encouraged to include photographs of the sampled tissues, either in the main manuscript or as supplementary material, to clearly document the tissue sampling strategy and enhance the reproducibility of the study.

**Discussion-**

- The manuscript requires extensive English language editing. Numerous grammatical mistakes, awkward phrasing (eg. his research conducted a comprehensive genome-wide identification), inconsistent tense usage, and poor sentence construction are present throughout the manuscript, making several sections difficult to read.
- The authors extensively discuss the regulatory roles of MeJA and ABA despite the absence of any experimental validation, such as hormone treatment assays or expression analyses following MeJA/ABA application. Since cis-regulatory element prediction only suggests potential regulatory mechanisms, the current discussion appears speculative and should be revised accordingly.
- The last paragraph of the Discussion is largely repetitive and overlaps substantially with the Conclusion section. It should be removed or significantly shortened to eliminate redundancy and improve the manuscript's readability.

Several references are not formatted according to the journal's guidelines. Please carefully check and correct the formatting of all references to ensure consistency throughout the reference list.

The figure legends should be more comprehensive. They should include sufficient methodological and explanatory details so that each figure can be understood without referring extensively to the main text.
