# Supplementary material for: Comprehensive Analysis of the AP2/ERF Transcription Factor Family in Schisandra chinensis and Its Implications for secondary metabolism
Source: PLoS One. 2026 Oct 15;21(10):e0361017. doi: 10.1371/journal.pone.0361017 (PMC13671533; doi:10.1371/journal.pone.0361017)
Supplement: Peer review history

# Peer review: Decision Letter 0

**DOI:** 10.1371/journal.pone.0361017.r001

**Authors:** Anil Kumar Singh

5 Aug 2026

Dear Dr. NIE,

Thank you for submitting your manuscript to PLOS ONE. After careful consideration, we feel that it has merit but does not fully meet PLOS ONE’s publication criteria as it currently stands. Therefore, we invite you to submit a revised version of the manuscript that addresses the points raised during the review process.

Please submit your revised manuscript by Oct 04 2026 11:59PM. If you will need more time than this to complete your revisions, please reply to this message or contact the journal office at plosone@plos.org. When you're ready to submit your revision, log on to https://www.editorialmanager.com/pone/ and select the 'Submissions Needing Revision' folder to locate your manuscript file.

- A letter that responds to each point raised by the academic editor and reviewer(s). You should upload this letter as a separate file labeled 'Response to Reviewers'.
- A marked-up copy of your manuscript that highlights changes made to the original version. You should upload this as a separate file labeled 'Revised Manuscript with Track Changes'.
- An unmarked version of your revised paper without tracked changes. You should upload this as a separate file labeled 'Manuscript'.

If you would like to make changes to your financial disclosure, please include your updated statement in your cover letter. Guidelines for resubmitting your figure files are available below the reviewer comments at the end of this letter.

If applicable, we recommend that you deposit your laboratory protocols in protocols.io to enhance the reproducibility of your results. Protocols.io assigns your protocol its own identifier (DOI) so that it can be cited independently in the future. For instructions see: https://journals.plos.org/plosone/s/submission-guidelines#loc-laboratory-protocols. Additionally, PLOS ONE offers an option for publishing peer-reviewed Lab Protocol articles, which describe protocols hosted on protocols.io. Read more information on sharing protocols at https://plos.org/protocols?utm_medium=editorial-email&utm_source=authorletters&utm_campaign=protocols.

As the corresponding author, your ORCID iD is verified in the submission system and will appear in the published article. PLOS supports the use of ORCID, and we encourage all coauthors to register for an ORCID iD and use it as well. Please encourage your coauthors to verify their ORCID iD within the submission system before final acceptance, as unverified ORCID iDs will not appear in the published article. *Only*  the individual author can complete the verification step; PLOS staff *cannot*  verify ORCID iDs on behalf of authors.

We look forward to receiving your revised manuscript.

Kind regards,

Anil Kumar Singh, Ph.D.

Academic Editor

PLOS One

Journal Requirements:

When submitting your revision, we need you to address these additional requirements.

1.Please ensure that your manuscript meets PLOS ONE's style requirements, including those for file naming. The PLOS ONE style templates can be found at

https://journals.plos.org/plosone/s/file?id=wjVg/PLOSOne_formatting_sample_main_body.pdf and

https://journals.plos.org/plosone/s/file?id=ba62/PLOSOne_formatting_sample_title_authors_affiliations.pdf

2. PLOS requires an ORCID iD for the corresponding author in Editorial Manager on papers submitted after December 6th, 2016. Please ensure that you have an ORCID iD and that it is validated in Editorial Manager. To do this, go to ‘Update my Information’ (in the upper left-hand corner of the main menu), and click on the Fetch/Validate link next to the ORCID field. This will take you to the ORCID site and allow you to create a new iD or authenticate a pre-existing iD in Editorial Manager.

3. Thank you for stating the following financial disclosure:

“Science and Technology Program of Jiangsu Provincial Administration for Market Regulation (KJ2025008)”

Please state what role the funders took in the study.  If the funders had no role, please state: "The funders had no role in study design, data collection and analysis, decision to publish, or preparation of the manuscript."

If this statement is not correct you must amend it as needed.

Please include this amended Role of Funder statement in your cover letter; we will change the online submission form on your behalf.

4. Please note that funding information should not appear in any section or other areas of your manuscript. We will only publish funding information present in the Funding Statement section of the online submission form. Please remove any funding-related text from the manuscript.

5. Thank you for stating the following in the Financial Disclosure section:

“Science and Technology Program of Jiangsu Provincial Administration for Market Regulation (KJ2025008)”

We note that one or more of the authors are employed by a commercial company: “Fujian Pien Tze Huang Cosmetics Co., Ltd.”

1. Please provide an amended Funding Statement declaring this commercial affiliation, as well as a statement regarding the Role of Funders in your study. If the funding organization did not play a role in the study design, data collection and analysis, decision to publish, or preparation of the manuscript and only provided financial support in the form of authors' salaries and/or research materials, please review your statements relating to the author contributions, and ensure you have specifically and accurately indicated the role(s) that these authors had in your study. You can update author roles in the Author Contributions section of the online submission form.

Please also include the following statement within your amended Funding Statement.

“The funder provided support in the form of salaries for authors ”YH, YS” but did not have any additional role in the study design, data collection and analysis, decision to publish, or preparation of the manuscript. The specific roles of these authors are articulated in the ‘author contributions’ section.”

If your commercial affiliation did play a role in your study, please state and explain this role within your updated Funding Statement.

2. Please also provide an updated Competing Interests Statement declaring this commercial affiliation along with any other relevant declarations relating to employment, consultancy, patents, products in development, or marketed products, etc.

Within your Competing Interests Statement, please confirm that this commercial affiliation does not alter your adherence to all PLOS ONE policies on sharing data and materials by including the following statement: "This does not alter our adherence to  PLOS ONE policies on sharing data and materials.” (as detailed online in our guide for authors http://journals.plos.org/plosone/s/competing-interests) . If this adherence statement is not accurate and  there are restrictions on sharing of data and/or materials, please state these. Please note that we cannot proceed with consideration of your article until this information has been declared.

Please include both an updated Funding Statement and Competing Interests Statement in your cover letter. We will change the online submission form on your behalf.

6. Please upload a new copy of Figure 1-7 as the detail is not clear. Please follow the link for more information:  https://journals.plos.org/plosone/s/figures

7. If the reviewer comments include a recommendation to cite specific previously published works, please review and evaluate these publications to determine whether they are relevant and should be cited. There is no requirement to cite these works unless the editor has indicated otherwise.

[Note: HTML markup is below. Please do not edit.]

Reviewer's Responses to Questions

**Comments to the Author**

1. Is the manuscript technically sound, and do the data support the conclusions?

Reviewer #1: Yes

Reviewer #2: Partly

**********

2. Has the statistical analysis been performed appropriately and rigorously?

Reviewer #1: Yes

Reviewer #2: N/A

**********

3. Have the authors made all data underlying the findings in their manuscript fully available?

The PLOS Data policy

Reviewer #1: Yes

Reviewer #2: Yes

**********

4. Is the manuscript presented in an intelligible fashion and written in standard English?

Reviewer #1: Yes

Reviewer #2: No

**********

Reviewer #1: Dear authors

This study presents a comprehensive genome-wide identification and characterization of the AP2/ERF transcription factor superfamily in Schisandra chinensis, a medicinal plant valued for its dibenzocyclooctadiene lignans with diverse pharmacological properties. Using the high-quality chromosome-level genome, the authors identified 144 ScAP2/ERF genes and classified them into five subfamilies (ERF, DREB, AP2, RAV, and Soloist) through phylogenetic analysis with Arabidopsis thaliana. Conserved motif and domain analyses confirmed the characteristic AP2 domain architecture across all members, with AP2 subfamily members containing two AP2 domains and RAV members possessing both AP2 and B3 domains. Chromosomal localization revealed uneven distribution across 14 chromosomes with distinct clusters on chromosomes 1, 2, 6, 10, 11, and 13, while synteny analysis identified 26 segmentally duplicated gene pairs within S. chinensis and 39 orthologous pairs with A. thaliana. Promoter cis-acting element analysis demonstrated enrichment of light-responsive, MeJA-responsive, ABA-responsive, and anaerobic induction elements, suggesting complex hormonal and environmental regulation. Expression profiling across roots, stems, leaves, and fruits revealed distinct tissue-specific patterns, with ten genes (including ScERF100, ScERF107, ScERF40, and ScERF94) showing notably high expression specifically in fruit tissues, which is the designated medicinal part according to the Chinese Pharmacopoeia. These fruit-specific genes may play potential regulatory roles in lignan biosynthesis and accumulation, as lignans such as schisandrol A, gomisin A, and schisandrin A are predominantly enriched in fruits. The study establishes a foundational framework for understanding the evolutionary history and functional diversification of the AP2/ERF family in S. chinensis and provides candidate genes for future functional validation. However, the study is entirely descriptive, lacking any functional experiments (e.g., hairy root transformation, promoter activity assays, or MeJA treatment) to validate the proposed regulatory roles, and all conclusions about lignan biosynthesis regulation remain correlative. Missing figures and the absence of Ka/Ks analysis for duplicated gene pairs are additional limitations that should be addressed in revision.

Abstract

- Claims "potential regulatory roles in lignan accumulation" but no functional validation provided – purely correlative

- Overstates "first comprehensive genome-wide identification" – novelty is limited to species-specific analysis, not methodological

- No quantitative data presented (gene numbers only, no expression fold-changes or statistical significance)

- "Candidate genes for subsequent functional validation" – acknowledges lack of functional data

Introduction

- Too long and contains excessive background on Schisandra medicinal properties and lignan biosynthesis (over 40 lines); could be condensed by 50%

- Research gap is stated but not clearly justified – why AP2/ERF specifically for lignan regulation rather than other TF families?

- No hypothesis explicitly stated

- Connection between AP2/ERF and lignan biosynthesis is weakly established – only three examples from other species, none in Schisandra

- References for lignan pathway are old (pre-2020); recent advances in Schisandra genomics not adequately cited

Methods

- Plant material: single collection site only – findings may not be generalizable

- No information on sample size per tissue for RNA-seq validation (only "three biological replicates" but no details)

- HMMER threshold (E-value 1e-") is incomplete – should specify numeric threshold (e.g., 1e-5 or 1e-10)

- No validation of RNA-seq data with qRT-PCR for all 122 expressed genes – only 10 genes validated (8.2%)

- qRT-PCR method: no mention of efficiency testing for each primer pair

- No negative controls for qRT-PCR (no-template controls mentioned but not in methods)

- Transcriptome data accession provided but no statement on data availability for genome files

Results

- Section 3.1 (physicochemical properties) is overly descriptive and could be condensed or moved to supplementary

- Instability index and GRAVY values reported but no biological interpretation – why do these matter?

- Figure 1 (phylogenetic tree) referenced but not included in text – reader cannot verify classification

- Chromosomal localization: "clustered distribution suggests gene duplication" – speculative without duplication time estimation (Ka/Ks)

- Synteny analysis: 26 pairs identified but no Ka/Ks ratio calculation – cannot determine if duplications are under purifying or positive selection

- Promoter analysis: 25 elements identified but only top 4 discussed – what about the other 21?

- Expression profiles: only 122 of 144 genes expressed in at least one tissue – what about the 22 genes with no expression?

- Fruit-specific genes: ten identified but no functional annotation or pathway mapping for these specific genes

- Figure 6 referenced but not included – missing key data

Discussion

- Discussion is too long and largely repeats results rather than interpreting them

- Claim "comparable to other species" (lines 334-341) is superficial – no critical comparison of functional differences

- Speculative statements about "light signaling pathways" without experimental evidence – correlation does not prove regulation

- MeJA-responsive elements discussed extensively but no MeJA treatment experiments were performed

- No discussion of limitations: single tissue collection site, no functional validation, no field or stress treatment data

- Comparison with other species (Salvia, Panax, Eschscholzia) is superficial – no discussion of why Schisandra might differ

- No discussion of the 22 genes with no expression – are they pseudogenes or stress-specific?

- No comparison with previous AP2/ERF studies in other medicinal plants beyond general statements

- Limitations not discussed at all (despite being a major weakness)

Conclusion

- Overclaims: "comprehensive genome-wide analysis" – descriptive only, no functional data

- "Establish a fundamental resource" – acknowledges study is descriptive

- "Provide candidate genes" – no functional validation, only correlation

- No specific genes prioritized for future study beyond listing the ten fruit-specific ones

- No mention of limitations or future validation needed

Other comments:

- No functional validation – all claims of regulatory roles in lignan biosynthesis are correlative

- Descriptive study only – genome-wide identification, phylogenetics, expression analysis – no functional experiments

- No validation experiments – no transient expression, no hairy root transformation, no promoter activity assays, no Y1H

- Missing figures – Figures 1, 4, 5, 6 referenced but not provided in text

- Data availability – transcriptome data accession provided but genome data accessibility not clearly stated

- Discussion too long – unnecessarily repeats results without synthesis

- References – some key citations (like the Schisandra genome paper) are not cited

- Results section overly descriptive – reads like a genome paper without functional insight

Reviewer #2: The manuscript presents a comprehensive study involving genome-wide identification, characterization, subcellular localization, phylogenetic analysis, and other aspects of the AP2/ERF gene family in Schisandra chinensis along with the tissue specific expression profiling. However, the manuscript lacks a clear rationale and explanation of the study's importance.

In conclusion, I recommend resubmitting the manuscript for further consideration. Prior to resubmission, the identified issues and recommended improvements are outlined below:

The inclusion of "lignan biosynthesis" in the title is not adequately supported by the data presented in the manuscript. No comprehensive analysis or functional investigation of lignan biosynthesis is provided throughout the study. The authors should revise the title to accurately reflect the actual scope and findings of the work.

Introduction-

• The introduction lacks a coherent scientific storyline. The authors should reorganize the introduction to build a stronger rationale leading to the central objective of the study.

• A substantial portion of the introduction is devoted to background information that does not directly contribute to the research objective. This results in an unnecessarily lengthy introduction and distracts the reader from the main scientific question.

• The introduction fails to adequately justify why AP2/ERF transcription factors was chosen for investigation. A stronger mechanistic rationale supported by previous studies is needed.

Materials and Methods-

• The formatting of botanical names is inconsistent throughout the manuscript. All scientific names of plant species should be written in italics. in accordance with standard taxonomic conventions. Please check and correct this throughout the manuscript.

• Please clarify the rationale for constructing the phylogenetic tree using only Arabidopsis thaliana and the study species. A phylogenetic tree based on only two species provides limited evolutionary insight and is generally insufficient to support evolutionary inferences. Inclusion of homologs from additional representative species would substantially strengthen the phylogenetic analysis.

• The cis-regulatory element analysis appears to rely on conventional prediction resources. The authors are encouraged to explore and compare results with more recent and comprehensive plant cis-regulatory databases or motif resources to improve the reliability and biological relevance of the predicted regulatory elements.

• Please justify the rationale for using Arabidopsis thaliana as the reference species for the synteny analysis.

• The authors have analyzed the tissue-specific expression pattern of AP2/ERF genes; however, it is unclear how these expression profiles were associated with lignan biosynthesis. Were lignan contents or compositions quantified in the corresponding tissues?

• AP2/ERF transcription factors constitute a large and functionally diverse family involved in numerous biological processes, including plant growth, development, and responses to various biotic and abiotic stresses. Therefore, tissue-specific expression alone is insufficient to infer their specific role in lignan biosynthesis.

• The qRT-PCR validation appears to be based only on biological replicates. Please clarify whether technical replicates were performed.

• The manuscript does not specify the statistical methods used to analyze the qRT-PCR data. Please clearly describe the statistical software, tests employed and significance thresholds used for the analysis.

Results-

• The subcellular localization of the candidate protein(s) has been predicted only through computational approaches. Experimental validation, such as transient expression of GFP fusion constructs in Nicotiana benthamiana leaves, would substantially strengthen the results.

• Figure 2 is difficult to interpret as the multiple sequence alignment is not organized according to the AP2/ERF subfamilies identified in the phylogenetic tree. To improve clarity authors should rearrange the alignment by grouping sequences according to their respective subfamilies.

• Tissue-specific expression analysis is a key component of this study; however, no representative images of the plant tissues used for qRT-PCR are provided. The authors are encouraged to include photographs of the sampled tissues, either in the main manuscript or as supplementary material, to clearly document the tissue sampling strategy and enhance the reproducibility of the study.

Discussion-

• The manuscript requires extensive English language editing. Numerous grammatical mistakes, awkward phrasing (eg. his research conducted a comprehensive genome-wide identification), inconsistent tense usage, and poor sentence construction are present throughout the manuscript, making several sections difficult to read.

• The authors extensively discuss the regulatory roles of MeJA and ABA despite the absence of any experimental validation, such as hormone treatment assays or expression analyses following MeJA/ABA application. Since cis-regulatory element prediction only suggests potential regulatory mechanisms, the current discussion appears speculative and should be revised accordingly.

• The last paragraph of the Discussion is largely repetitive and overlaps substantially with the Conclusion section. It should be removed or significantly shortened to eliminate redundancy and improve the manuscript's readability.

Several references are not formatted according to the journal's guidelines. Please carefully check and correct the formatting of all references to ensure consistency throughout the reference list.

The figure legends should be more comprehensive. They should include sufficient methodological and explanatory details so that each figure can be understood without referring extensively to the main text.

**********

what does this mean?). If published, this will include your full peer review and any attached files.

If you choose “no”, your identity will remain anonymous but your review may still be made public.

**Do you want your identity to be public for this peer review?**  For information about this choice, including consent withdrawal, please see our Privacy Policy

Reviewer #1: **Yes:**  ALI MOGHADAM

Reviewer #2: No

**********

[NOTE: If reviewer comments were submitted as an attachment file, they will be attached to this email and accessible via the submission site. Please log into your account, locate the manuscript record, and check for the action link "View Attachments". If this link does not appear, there are no attachment files.]

To ensure your figures meet our technical requirements, please review our figure guidelines: https://journals.plos.org/plosone/s/figures

You may also use PLOS’s free figure tool, NAAS, to help you prepare publication quality figures: https://journals.plos.org/plosone/s/figures#loc-tools-for-figure-preparation.

NAAS will assess whether your figures meet our technical requirements by comparing each figure against our figure specifications.

Attachment Submitted filename: Comments for author plos one.docx

# Author comment: Author response to Decision Letter 1

**DOI:** 10.1371/journal.pone.0361017.r002

17 Sep 2026

Dear Editor and Reviewers,

Thank you very much for your time and constructive comments on our manuscript. We have carefully revised the manuscript according to all suggestions. Below is our point-by-point response, and all changes are marked in red. We hope the revised manuscript is now suitable for publication.

Journal Requirements:

When submitting your revision, we need you to address these additional requirements.

1.Please ensure that your manuscript meets PLOS ONE's style requirements, including those for file naming. The PLOS ONE style templates can be found at

https://journals.plos.org/plosone/s/file?id=wjVg/PLOSOne_formatting_sample_main_body.pdf and

https://journals.plos.org/plosone/s/file?id=ba62/PLOSOne_formatting_sample_title_authors_affiliations.pdf

Response: We have ensured that the revised manuscript complies with PLOS ONE style requirements, including file naming conventions.

2.PLOS requires an ORCID iD for the corresponding author in Editorial Manager on papers submitted after December 6th, 2016. Please ensure that you have an ORCID iD and that it is validated in Editorial Manager. To do this, go to ‘Update my Information’ (in the upper left-hand corner of the main menu), and click on the Fetch/Validate link next to the ORCID field. This will take you to the ORCID site and allow you to create a new iD or authenticate a pre-existing iD in Editorial Manager.

Response: The corresponding author’s ORCID iD has been validated in Editorial Manager.

Wei Nie ORCID iD: 0009-0001-9670-8032

3. Thank you for stating the following financial disclosure:

“Science and Technology Program of Jiangsu Provincial Administration for Market Regulation (KJ2025008)”

Please state what role the funders took in the study. If the funders had no role, please state: "The funders had no role in study design, data collection and analysis, decision to publish, or preparation of the manuscript."

If this statement is not correct you must amend it as needed.

Please include this amended Role of Funder statement in your cover letter; we will change the online submission form on your behalf.

Response: We have included the following Role of Funder statement in the updated Cover Letter: ‘The funders had no role in study design, data collection and analysis, decision to publish, or preparation of the manuscript.’

4.Please note that funding information should not appear in any section or other areas of your manuscript. We will only publish funding information present in the Funding Statement section of the online submission form. Please remove any funding-related text from the manuscript.

Response: We have removed the funding information from the manuscript body. Funding information is now provided only in the online submission form.

5. Thank you for stating the following in the Financial Disclosure section:

“Science and Technology Program of Jiangsu Provincial Administration for Market Regulation (KJ2025008)”

We note that one or more of the authors are employed by a commercial company: “Fujian Pien Tze Huang Cosmetics Co., Ltd.”

1. Please provide an amended Funding Statement declaring this commercial affiliation, as well as a statement regarding the Role of Funders in your study. If the funding organization did not play a role in the study design, data collection and analysis, decision to publish, or preparation of the manuscript and only provided financial support in the form of authors' salaries and/or research materials, please review your statements relating to the author contributions, and ensure you have specifically and accurately indicated the role(s) that these authors had in your study. You can update author roles in the Author Contributions section of the online submission form.

Please also include the following statement within your amended Funding Statement.

“The funder provided support in the form of salaries for authors ”YH, YS” but did not have any additional role in the study design, data collection and analysis, decision to publish, or preparation of the manuscript. The specific roles of these authors are articulated in the ‘author contributions’ section.”

If your commercial affiliation did play a role in your study, please state and explain this role within your updated Funding Statement.

6. Please also provide an updated Competing Interests Statement declaring this commercial affiliation along with any other relevant declarations relating to employment, consultancy, patents, products in development, or marketed products, etc.

Within your Competing Interests Statement, please confirm that this commercial affiliation does not alter your adherence to all PLOS ONE policies on sharing data and materials by including the following statement: "This does not alter our adherence to PLOS ONE policies on sharing data and materials.” (as detailed online in our guide for authors http://journals.plos.org/plosone/s/competing-interests) . If this adherence statement is not accurate and there are restrictions on sharing of data and/or materials, please state these. Please note that we cannot proceed with consideration of your article until this information has been declared.

Please include both an updated Funding Statement and Competing Interests Statement in your cover letter. We will change the online submission form on your behalf.

Response: We have included the following amended Funding Statement in the Cover Letter: ‘The funder provided support in the form of salaries for authors YH and YS but did not have any additional role in the study design, data collection and analysis, decision to publish, or preparation of the manuscript. The specific roles of these authors are articulated in the ‘author contributions’ section.’ We have also included an updated Competing Interests Statement in the Cover Letter.

7.Please upload a new copy of Figure 1-7 as the detail is not clear. Please follow the link for more information: https://journals.plos.org/plosone/s/figures

Response: We have uploaded new, higher-resolution versions of Figures 1–7 with improved clarity and detail, following the PLOS ONE figure guidelines.

8. If the reviewer comments include a recommendation to cite specific previously published works, please review and evaluate these publications to determine whether they are relevant and should be cited. There is no requirement to cite these works unless the editor has indicated otherwise.

Response: We have reviewed the suggested publications and evaluated their relevance. We have added recent and relevant references where appropriate throughout the revised manuscript.

Comments to the Author

Please use the space provided to explain your answers to the questions above. You may also include additional comments for the author, including concerns about dual publication, research ethics, or publication ethics. (Please upload your review as an attachment if it exceeds 20,000 characters)

Reviewer #1: Dear authors

This study presents a comprehensive genome-wide identification and characterization of the AP2/ERF transcription factor superfamily in Schisandra chinensis, a medicinal plant valued for its dibenzocyclooctadiene lignans with diverse pharmacological properties. Using the high-quality chromosome-level genome, the authors identified 144 ScAP2/ERF genes and classified them into five subfamilies (ERF, DREB, AP2, RAV, and Soloist) through phylogenetic analysis with Arabidopsis thaliana. Conserved motif and domain analyses confirmed the characteristic AP2 domain architecture across all members, with AP2 subfamily members containing two AP2 domains and RAV members possessing both AP2 and B3 domains. Chromosomal localization revealed uneven distribution across 14 chromosomes with distinct clusters on chromosomes 1, 2, 6, 10, 11, and 13, while synteny analysis identified 26 segmentally duplicated gene pairs within S. chinensis and 39 orthologous pairs with A. thaliana. Promoter cis-acting element analysis demonstrated enrichment of light-responsive, MeJA-responsive, ABA-responsive, and anaerobic induction elements, suggesting complex hormonal and environmental regulation. Expression profiling across roots, stems, leaves, and fruits revealed distinct tissue-specific patterns, with ten genes (including ScERF100, ScERF107, ScERF40, and ScERF94) showing notably high expression specifically in fruit tissues, which is the designated medicinal part according to the Chinese Pharmacopoeia. These fruit-specific genes may play potential regulatory roles in lignan biosynthesis and accumulation, as lignans such as schisandrol A, gomisin A, and schisandrin A are predominantly enriched in fruits. The study establishes a foundational framework for understanding the evolutionary history and functional diversification of the AP2/ERF family in S. chinensis and provides candidate genes for future functional validation. However, the study is entirely descriptive, lacking any functional experiments (e.g., hairy root transformation, promoter activity assays, or MeJA treatment) to validate the proposed regulatory roles, and all conclusions about lignan biosynthesis regulation remain correlative. Missing figures and the absence of Ka/Ks analysis for duplicated gene pairs are additional limitations that should be addressed in revision.

Abstract

- Claims "potential regulatory roles in lignan accumulation" but no functional validation provided – purely correlative

Response: We thank the reviewer for this important comment and agree that, in the absence of functional validation, the proposed regulatory roles remain correlative. We have revised the abstract accordingly to avoid any overstatement: the original wording has been replaced with "suggesting their potential regulatory roles in the accumulation of pharmacologically active compounds in the medicinal fruit," where the terms "suggesting" and "potential" explicitly frame these inferences as hypotheses rather than demonstrated functions. In addition, we have added a limitations paragraph at the end of the Discussion, in which we explicitly acknowledge that the regulatory roles proposed for ScAP2/ERF genes are inferred from computational identification and expression profiling rather than from experimental functional validation (e.g., hairy root transformation, promoter activity assays, or hormone treatment analyses), and therefore remain correlative pending such confirmation. We believe the revised text now accurately reflects the evidence level of this study.

- Overstates "first comprehensive genome-wide identification" – novelty is limited to species-specific analysis, not methodological.

Response: We agree with the reviewer that the novelty of this work lies in the species-specific comprehensive characterization rather than in methodological innovation, and we have removed the priority claim accordingly. The sentence now reads: "This study represents a comprehensive genome-wide identification of the AP2/ERF family in S. chinensis, thereby establishing a foundational framework for understanding its evolutionary history and functional diversification." We have framed the contribution of this work as providing the first systematic, chromosome-level characterization of this gene family in S. chinensis and a foundational resource for the community, rather than as a methodological advance.

- No quantitative data presented (gene numbers only, no expression fold-changes or statistical significance).

Response: We thank the reviewer for this suggestion and have substantially expanded the quantitative content of the manuscript in response. First, in the abstract we now report that "Of the 144 identified genes, 122 (84.7%) were detectably expressed in at least one tissue," and we specify the tissues profiled (roots, stems, leaves, and fruits), thereby grounding the tissue-specificity statements in concrete data. Second, to address the concern regarding statistical significance, we have added a new supplementary table (S4 Table) that reports, for each of the 122 expressed ScAP2/ERF genes, (i) the mean FPKM values in each of the six tissues (tip stem, mature stem, tip leaf, old leaf, fruit, and root), calculated from three biological replicates per tissue, and (ii) the statistical significance of expression differences across tissues, assessed by one-way ANOVA (p-value). Because this study compares expression across multiple tissues rather than between two conditions, one-way ANOVA — rather than pairwise fold-change — is the appropriate statistical test for identifying genes with significant tissue-specific expression. The mean FPKM values provided in S3 Table also allow readers to directly quantify expression magnitude and inter-tissue differences; fold differences between any two tissues can be readily derived from the reported FPKM values.

- "Candidate genes for subsequent functional validation" – acknowledges lack of functional data.

Response: We agree that this study does not include functional data, and we have revised the sentence to make this scope explicit: "Furthermore, it offers candidate genes for subsequent functional validation of their potential roles in regulating secondary metabolism." We have retained this statement because genome-wide identification studies are inherently positioned to generate characterized gene resources and testable hypotheses for subsequent functional work; presenting the identified genes as candidates for future validation, rather than as functionally characterized regulators, is consistent with this study design and with comparable genome-wide analyses in other medicinal plant species[1-2]. The absence of functional validation is now transparently acknowledged in the newly added limitations paragraph in the Discussion, which also outlines the specific experimental approaches (e.g., yeast one-hybrid, dual-luciferase reporter, and transient expression assays) planned for future validation.

[1] Hu F, Zhang Y, Guo J. Identification and Characterization of AP2/ERF Transcription Factors in Yellow Horn. Int J Mol Sci. 2022 Nov 30;23(23) pii: ijms232314991. doi: 10.3390/ijms232314991. PubMed PMID: 36499319;

[2] Shing P, Islam MSU, Khatun MS, Zohra FT, Hasan N, Rahman SM, Sarkar MAR. Genome-wide identification, characterization and expression profiles of FORMIN gene family in cotton (Gossypium Raimondii L.). BMC Genom Data. 2024 Dec 18;25(1):105. pii: 10.1186/s12863-024-01285-z. doi: 10.1186/s12863-024-01285-z. PubMed PMID: 39695391.

Introduction

- Too long and contains excessive background on Schisandra medicinal properties and lignan biosynthesis (over 40 lines); could be condensed by 50%.

Response: We have substantially condensed the Introduction, reducing it by approximately 40%. The detailed description of medicinal properties and lignan biosynthetic pathway has been streamlined into a single paragraph, allowing the focus to shift more quickly to the AP2/ERF family and the study objectives.

- Research gap is stated but not clearly justified – why AP2/ERF specifically for lignan regulation rather than other TF families?

Response: We have added a clearer justification for focusing on the AP2/ERF family, citing specific examples where AP2/ERF transcription factors have been functionally linked to secondary metabolite biosynthesis in medicinal plants (SmERF1L1 in S. miltiorrhiza, PgERF120 in P. ginseng, Ii049 in I. indigotica). These examples establish the AP2/ERF family as a functionally versatile group with demonstrated roles in regulating medicinal compound biosynthesis, providing strong rationale for our study.

- No hypothesis explicitly stated.

Response: We have added an explicit hypothesis statement in the revised Introduction: ‘Given that AP2/ERF members have been functionally linked to lignan and other specialized metabolic pathways in related species, we hypothesized that the AP2/ERF family in S. chinensis may include members that potentially regulate DBCOD lignan biosynthesis, and that their tissue-specific expression patterns may correlate with the differential accumulation of lignans across organs.’

- Connection between AP2/ERF and lignan biosynthesis is weakly established – only three examples from other species, none in Schisandra.

Response: We acknowledge that direct evidence linking AP2/ERF to lignan biosynthesis in Schisandra is currently lacking. We have revised the text to present the examples from other species as providing a rationale for our investigation, rather than as direct evidence. We have also clarified that our study aims to identify candidate regulators through computational and transcriptomic approaches, with functional validation as an important future direction.

- References for lignan pathway are old (pre-2020); recent advances in Schisandra genomics not adequately cited.

Response: We thank the reviewer for this comment. We have updated the Introduction with recent literature, including the newly published chromosome-level genome of S. chinensis (Liu et al., 2025, Science Advances), which reports lineage-specialized lignan biosynthesis in Schisandra. In addition, we cited Ushimaru (2024) on the enzymatic mechanisms of lignan assembly and Choi et al. (2026) on metabolic engineering strategies for lignan production. These references now support all statements on the lignan pathway and Schisandra genomics in the Introduction.

[1]. Ushimaru R. Functions and mechanisms of enzymes assembling lignans and norlignans. Curr Opin Chem Biol. 2024;80:102462. http://doi.org/10.1016/j.cbpa.2024.102462

[2]. Liu J, Xiong R, Liu L, Zhou T, Xue J, Sun D, et al. Insights into angiosperm evolution and lineage-specialized lignan biosynthesis from the early-diverging Schisandra genome. Sci Adv. 2025;11(33):eadw486. http://doi.org/10.1126/sciadv.adw0486

[3]. Choi S, Lee SB, Kim B. Metabolic engineering strategies for optimized lignan production in plants. Front Plant Sci. 2026;17:1827862. http://doi.org/10.3389/fpls.2026.1827862

Methods

- Plant material: single collection site only – findings may not be generalizable.

Response: We thank the reviewer for this comment and acknowledge that plant materials were collected from a single location. However, the aim of this study was the genome-wide identification of the AP2/ERF gene family and tissue-specific expression profiling in S. chinensis, rather than an investigation of geographic variation or population-level generalizability. For such basic genomic and expression analyses, materials from a single representative site are sufficient, and this is consistent with the standard practice in comparable genome-wide gene family studies in other plant species [1-3]. We agree that future studies incorporating samples from multiple geographic locations would further strengthen the findings, particularly regarding natural variation in expression pattern.

[1] Hu F, Zhang Y, Guo J. Identification and Characterization of AP2/ERF Transcription Factors in Yellow Horn. Int J Mol Sci. 2022 Nov 30;23(23) pii: ijms232314991. doi: 10.3390/ijms232314991. PubMed PMID: 36499319;

[2] Shing P, Islam MSU, Khatun MS, Zohra FT, Hasan N, Rahman SM, Sarkar MAR. Genome-wide identification, characterization and expression profiles of FORMIN gene family in cotton (Gossypium Raimondii L.). BMC Genom Data. 2024 Dec 18;25(1):105. pii: 10.1186/s12863-024-01285-z. doi: 10.1186/s12863-024-01285-z. PubMed PMID: 39695391.

[3] Sun M, Li J, Zhang Y, Tian L, Miao Y, Bai L, Hou L. Genome-wide identification of tomato B-RRs gene family and expression analysis in response to ABA and drought. PLoS One. 2026;21(8):e0356487. pii: PONE-D-25-29384. doi: 10.1371/journal.pone.0356487. PubMed PMID: 42607067.

- No information on sample size per tissue for RNA-seq validation (only "three biological replicates" but no details).

Response: We have clarified in the revised Methods that each biological replicate consisted of pooled tissues from three individual plants, and that three biological replicates were used for each tissue type. This information has been added to Section 2.1.

- HMMER threshold (E-value 1e-") is incomplete – should specify numeric threshold (e.g., 1e-5 or 1e-10).

Response: We apologize for this typographical error. The E-value threshold was 1e−5, which has been corrected in the revised Methods section.

- No validation of RNA-seq data with qRT-PCR for all 122 expressed genes – only 10 genes validated (8.2%).

Response: We thank the reviewer for this comment. Full qRT-PCR validation of all 122 expressed genes would indeed be more comprehensive; however, our validation was designed to verify the reliability of the transcriptomic data for the most biologically relevant candidates. We therefore prioritized the ten fruit-specific highly expressed genes, which are the core candidates for potential regulatory roles in fruit-specific compound accumulation and the focus of future functional studies. This targeted validation strategy is consistent with the standard practice in comparable genome-wide gene family studies, in which key candidate genes rather than the entire family are selected for qRT-PCR validation [1-2]. Moreover, the strong concordance between RNA-seq and qRT-PCR results for these ten genes (Figure 7B) supports the overall reliability of the transcriptomic data.

[1]Lei L, Dong K, Liu S, Li Y, Xu G, Sun H. Genome-wide identification of the WRKY gene family in blueberry (Vaccinium spp.) and expression analysis under abiotic stress. Front Plant Sci. 2024;15:1447749. doi: 10.3389/fpls.2024.1447749. PubMed PMID: 39211844.

[2]Li S, Ji Q, An X, Chen C, Luo X, Liu T, Zou L. Genome-wide analysis of WRKY gene family and the dynamic responses of key WRKY genes involved in cadmium stress in Brassica juncea. Front Plant Sci. 2024;15:1465905. doi: 10.3389/fpls.2024.1465905. PubMed PMID: 39450073.

- qRT-PCR method: no mention of efficiency testing for each primer pair;

Response: We have added information about primer amplification efficiency in the revised Methods. Primer efficiency was verified using a five-point standard curve (R² > 0.99), yielding efficiencies between 90% and 110% for all primer pairs.

- No negative controls for qRT-PCR (no-template controls mentioned but not in methods);

Response: We have added clarification that no-template controls (NTC) were included for each primer pair to rule out contamination. This has been specified in the revised Methods section.

- Transcriptome data accession provided but no statement on data availability for genome files

Response: We clarify that both the reference genome assembly and the raw transcriptome data used in this study were obtained from the previously published high-quality S. chinensis genome (Liu et al., 2025, Science Advances), and no new sequencing data were generated in the present work. All of these data have been deposited in publicly accessible databases by the original authors (Genome Sequence Archive accession no. CRA019045; BioProject PRJCA030150) and are freely available to all researchers. In the method, we described the source of the data and cited the reference of S. chinensis genome (Liu et al., 2025, Science Advances).

“Transcriptome data for various S. chinensis tissues (roots, stems, leaves, and fruits) were retrieved from the Beijing Institute of Genomics (Chinese Academy of Sciences) Big Data Center (accession: CRA019045 / PRJCA030150) [8].”

Results

- Section 3.1 (physicochemical properties) is overly descriptive and could be condensed or moved to supplementary

Response: We have condensed Section 3.1 by removing redundant details and focusing on the most informative findings. Additionally, we have added biological interpretation for the instability index and GRAVY values, explaining their relevance to transcription factor function.

- Instability index and GRAVY values reported but no biological interpretation – why do these matter?

Response: We have added biological interpretation in the revised Results. Specifically, we note that the negative GRAVY values are consistent with the hydrophilic nature of transcription factors functioning in aqueous nuclear environments, and that proteins with instability indices above 40 may undergo rapid turnover, potentially facilitating dynamic transcriptional responses.

- Figure 1 (phylogenetic tree) referenced but not included in text – reader cannot verify classification

Response: We thank the reviewer for this comment. Figure 1, which presents the phylogenetic tree of the AP2/ERF superfamily, is included in the revised manuscript. To help readers verify the classification directly from the figure, we have expanded the figure legend to fully describe the tree composition and annotation: the tree comprises 144 ScAP2/ERF proteins from S. chinensis (labeled with the "Sc" prefix) and 140 AtAP2/ERF proteins from A. thaliana (labeled with the "At" prefix), and all proteins are color-coded by subfamily — ERF (green), DREB (blue), AP2 (red), RAV (orange), and Soloist (purple). With this legend, the subfamily assignment of each gene can be traced directly in the figure, and the classification is consistent with the phylogenetic results reported in the Results section

- Chromosomal localization: "clustered distribution suggests gene duplication" – speculative without duplication time estimation (Ka/Ks)

Response: We sincerely thank the reviewer for this critical and highly constructive comment. We fully agree that chromosomal clustering alone is merely a structural observation and lacks the evolutionary dimension required to substantiate genuine duplication events.

To rigorously address this concern, we systematically dissected the 15 identified tandem chromosomal clusters into 40 adjacent gene pairs (representing successive duplication steps within each array) and performed comprehensive pairwise Ka/Ks-based evolutionary analysis. As fully detailed in the newly supplemented S3 Table. KaKs calculation of 26 gene pairs, our results not only confirm the occurrence of tandem duplications but also reveal a previously unrecognized temporal hierarchy among these events. Based on the estimated Ks values and substitution saturation patterns, we categorized the duplication evidence into three well-defined tiers:

1.Recent duplications with reliable Ks estimates (quantifiable timing):

For 8 adjacent pairs (e.g., Chr8: ScERF84/85 [Ks=0.057, Ka/Ks=0.728]; Chr8: ScERF85/86 [Ks=0.313, Ka/Ks=0.710]; Chr10: ScERF64/65 [Ks=0.039, Ka/Ks=0.112]; and the Chr14 cluster pairs with Ks ranging from 0.359 to 0.573), we obtained robust Ks values that explicitly confirm recent duplications with measurable divergence timelines.

2.Ultra-recent duplications with zero synonymous substitutions (the strongest line of evidence):

Notably, two adjacent pairs—ScERF20/21 (Chr10) and ScERF125/126 (Chr11)—exhibited zero synonymous substitutions (pS = 0). This indicates that these duplicate pairs are virtually identical and have emerged so recently that no neutral synonymous mutations have had time to accumulate since their duplication. This provides unequivocal, direct support for de novo tandem duplication events.

3.Ancient duplicated relics with saturated synonymous substitutions (pS ≥ 0.75)：

We acknowledge that Ks is not estimable for approximately half of the adjacent pairs (pS ≥ 0.75, marked as "saturation"). However, saturation does not invalidate the duplication hypothesis; rather, it is a hallmark of ancient tandem duplication events. Critically, the consistently low Ka/Ks values (or constrained pN/pS ratios) observed among these saturated pairs demonstrate that even these ancient duplicates have been preserved by strong purifying selection, rather than representing random genomic noise or assembly artifacts.

In conclusion, our pairwise Ka/Ks analysis provides robust, quantitative evolutionary evidence that unequivocally supports the tandem duplication origin of the clustered ScERF genes. The evidence spans from extremely recent (pS=0) to ancient (saturation), indicating that tandem expansion of this family has been a recurrent and ongoing process throughout sugarcane evolution.

Accordingly, we have revised the original speculative statement in the manuscript. The updated text now reads as follows:

To determine whether these clustered arrangements result from genuine duplication events, we performed pairwise Ka/Ks-based evolutionary analysis on all 40 adjacent gene pairs within the tandem arrays (S3 Table). The results confirmed that these clusters indeed originated from tandem duplications, with divergence times ranging from recent (e.g., Chr8: ScERF84/85, Ks = 0.057; Chr10: ScERF64/65, Ks = 0.039) to ancient (pS ≥ 0.75, Ks saturation), and even including ultra-recent pairs with zero synonymous substitutions (Chr10: ScERF20/21; Chr11: ScERF125/126). Notably, the consistently low Ka/Ks values across most evaluable pairs indicate that these duplicated copies have been predominantly maintained by purifying selection.

We believe this quantitative, data-driven revision thoroughly addresses the reviewer's concern and significantly strengthens the evolutionary interpretation of our chromosomal localization data. All relevant raw data and calculations have been added to the supplementary materials for full transparency.

- Synteny analysis: 26 pairs identified but no Ka/Ks ratio calculation – cannot determine if duplications are under purifying or positive selection.

Response: Synteny analysis within the S. chinensis genome was performed using MCScanX. The results revealed 26 pairs of ScAP2/ERF genes distributed across 14 chromosomes, mainly located on Chr2, Chr5, Chr6, Chr9, Chr10, and Chr11 (Figure 5A). These gene pairs are likely derived from gene duplication events and may therefore share similar biological functions. Gene duplication generally promotes mutations that can lead to novel functions or generate genes essential for plant adaptation to new environments.

Our results unequivocally resolve the selection pressure regimes governing these syntenic duplications. Among the 26 syntenic pairs, 25 yielded reliable Ka/Ks estimates, all of which are significantly less than 1 (ranging from 0.156 to 0.490). The remaining one pair (ScERF100/ScERF27) exhibited synonymous substitution saturation (pS ≥ 0.75, Ks not estimable), which is indicative of an ancient duplication event; nevertheless, its pN/pS ratio (0.449/0.778) similarly supports a pattern of non-neutral constraint.

Specifically, the Ka/Ks values for the evaluable pairs are as follows: ScERF79/96 (0.490, the highest observed value, yet still far below 1), ScERF143/16 (0.454), ScERF90/142 (0.381), ScERF136/134 (0.357), ScERF20/16 (0.353), ScERF03/10 (0.349), ScERF18/114 (0.334), ScERF135/137 (0.264), ScERF70/68 (0.261), ScERF33/31 (0.257), ScERF15/08 (0.246), ScERF04/02 (0.240), ScERF115/112 (0.235), ScERF19/06 (0.231), ScERF46/44 (0.229), ScERF66/48 (0.217, observed twice independently), ScERF130/140 (0.217), ScERF29/38 (0.209), ScERF117/114 (0.187), ScERF107/110 (0.157), and others with similarly low values.

These consistent Ka/Ks << 1 values unambiguously demonstrate that the majority of these syntenic duplicated ScAP2/ERF gene pairs have been subjected to strong purifying (negative) selection, rather than positive selection. This finding is biologically meaningful: it indicates that after whole-genome or segmental duplication events, these retained syntenic copies are functionally constrained and preserved to maintain genetic dosage balance and core regulatory functions, which is a typical evolutionary hallmark of transcription factor families in polyploid plants like sugarcane.

We have revised the manuscript accordingly to explicitly state this selection pressure conclusion in both the Results sections. The original speculative statement has been replaced with data-driven text:

To evaluate the selection pressure on these retained duplicates, we calculated the Ka/Ks ratios for all 26 pairs (S4 Table). Except for one saturated pair (ScERF100/ScERF27, pS ≥ 0.75), the remaining 25 pairs exhibited Ka/Ks values significantly below 1, ranging from 0.156 to 0.490, indicating that these syntenic duplicates are predominantly under purifying (negative) selection rather than positive selection. This suggests strong functional constraints and dosage balance maintenance after duplication.

We believe this comprehensive quantitative analysis thoroughly addresses the reviewer's concern and substantially strengthens the evolutionary interpretation of our synteny data.

- Promoter analysis: 25 elements identified but only top 4 discussed – what about the other 21?

Response: We thank the reviewer for this valid point. We agree that the original manuscript did not adequately present the full spectrum of the 25 cis-acting element types identified. In the revised main text, we have expanded the description of the remaining 21 elements by grouping them into functional categories rather than listing them individually, as many of these elements belong to the same broader regulatory pathways (e.g., hormone responses, stress responses, growth/development, and core promoter elements.

Hormone-responsive elements (beyond MeJA and ABA): including auxin-responsive elements, gibberellin-responsive elements, and salicylic acid-responsive elements;

Stress-responsive elements (beyond anaerobic induction and drought): including low-temperature-responsive elements, defense and stress-responsive elements , and wound-responsive elements;

Growth and development-related elements: including meristem expression-related elements, endosperm expression elements, and seed-specific regulation elements;

We have retained the focused discussion on the top four most abundant elements (light-responsive, MeJA-responsive, ABA-responsive, and anaerobic induction elements, accounting for 37%, 14%, 13%, and 10%, respectively), as these represent the predominant regulatory signals and are most relevant to the putative functions of ScAP2/ERF genes. Other elements are briefly mentioned in the text:

Other elements identified included auxin-responsive, gibberellin-responsive, salicylic acid-responsive, low-temperature-responsive, and wound-responsive elements, as well as meristem- and seed-specific expression elements, implying that the expression regulation of ScAP2/ERF genes is a complex process involving multiple environmental factors and hormonal signals.

- Expression profiles: only 122 of 144 genes expressed in at least one tissue – what about the 22 genes with no expression?

Response: We appreciate the reviewer’s attention to the 22 ScAP2/ERF genes showing no detectable expression. We fully acknowledge that gene expression is a dynamic process, and the RNA‑seq data merely represent a transcriptional snapshot under the specific tissues and growth conditions sampled. Thus, the absence of detectable transcripts does not necessarily indicate a lack of function or pseudogenization; rather, these genes may be expressed at other developmental stages, in unexamined tissues, or under specific stress conditions not covered in our current sampling. In addition, the possibility of extremely low transcript abundance or genuine loss of function in certain relaxed duplicates (as suggested by Ka/Ks evidence) cannot be excluded.

Discussion

- Discussion is too long and largely repeats results rather than interpreting them.

Response: We have substantially shortened the Discussion by removing repetitive descriptions of results and focusing on interpretation and comparison with other species. The revised Discussion is approximately 30% shorter than the original.

- Claim "comparable to other species" (lines 334-341) is superficial – no critical comparison of functional differences

Response: We have revised the comparative discussion to focus on specific patterns and their functional implications rather than simply comparing gene numbers. We now discuss why certain subfamilies predominate and what this means in the context of stress responses in terrestrial plants.

- Speculative statements about "light signaling pathways" without experimental evidence – correlation does not prove regulation.

Response: We have toned down the discussion of light signaling by noting that while the enrichment of light-responsive elements suggests potential involvement, direct experimental evidence is needed. The revised text presents this as a hypothesis for future testing rather than a conclusion.

- MeJA-responsive elements discussed extensively but no MeJA treatment experiments were performed.

Response: We thank the reviewer for pointing out this important limitation. We fully agree that discussing MeJA-responsive elements without experimental validation was speculative. To address this concern, we have performed additional MeJA treatment experiments on S. chinensis seedlings and revised the manuscript accordingly.

1. Supplementary MeJA treatment experiment

The detailed experimental procedure has been added to the Materials and Methods section:

“S. chinensis seedlings at a height of 20–30 cm were selected for hormone treatment. A 1 mM methyl jasmonate (MeJA) solution was prepared and applied to the plants using two routes: 100 mL was sprayed onto the foliar surface, and another 100 mL was drenched into the soil around the roots. Leaf tissues were collected at 0, 6, 12, 24, and 48 h post-treatment. Immediately after harvesting, the leaves were rinsed thoroughly with distilled water, blotted dry, and flash-frozen in liquid nitrogen. All samples were stored at −80°C until used for qRT-PCR analysis. Three independent biological replicates were performed for each time point.”

2. Expression analysis of the ten fruit-preferential ScERFs

We examined the expression of the ten fruit-preferentially expressed ScAP2/ERF genes (ScERF100, ScERF107, ScERF40, ScERF38, ScERF05, ScERF94, ScERF104, ScERF108, ScERF102, and ScERF64) in response to MeJA treatment. The results are presented in Figure 7. All ten genes were responsive to MeJA, exhibiting rapid and transient induction. Specifically, ScERF38, ScERF100, ScERF104, ScERF107, and ScERF108 peaked at 6 h, while ScERF05, ScERF40, ScERF64, and ScERF102 peaked at 12 h. These expression patterns are consistent with the presence of MeJA-responsive cis-elements in their promoters and provide direct experimental evidence for their MeJA responsiveness.

3. Revision of the Discussion

Based on these new data, we have revised the Discussion to remove speculative statements and to present the MeJA-related findings as experimentally supported observations. The revised text now clearly states that these ScAP2/ERF genes respond to MeJA at the transcriptional level, supporting their potential involvement in MeJA-mediated regulation of secondary metabolism. We have also toned down any overinterpretation regarding downstream regulatory functions, which will require further experimental validation.

- No discussion of limitations: single tissue collection site, no functional validation, no field or stress treatment data。

Response: We have added a dedicated limitations paragraph that addresses.

Conclusion

- Overclaims: "comprehensive genome-wide analysis" – descriptive only, no functional data.

- "Establish a fundamental resource" – acknowledges study is descriptive

- "Provide candidate genes" – no functional validation, only correlation

- No specific genes prioritized for future study beyond listing the ten fruit-specific ones

- No mention of limitations or future validation needed

Response: We have revised the Conclusion to use more measured language, added acknowledgment that ‘the present study is limited by its computational and descriptive nature,’ and emphasized the need for ‘future experimental investigation’.

Reviewer #2: The manuscript presents a comprehensive study involving genome-wide identification, characterization, subcellular localization, phylogenetic analysis, and other aspects of the AP2/ERF gene family in Schisandra chinensis along with the tissue specific expression profiling. However, the manuscript lacks a clear rationale and explanation of the study's importance.

In conclusion, I recommend resubmitting the manuscript for further consideration. Prior to resubmission, the identified issues and recommended improvements are outlined below:

The inclusion of "lignan biosynthesis" in the title is not adequately supported by the data presented in the manuscript. No comprehensive analysis or functional investigation of lignan biosynthesis is provided throughout the study. The authors should revise the title to accurately reflect the actual scope and findings of the work.

Response: We agree with this assessment. We have revised the title to ‘Comprehensive Analysis of the AP2/ERF Transcription Factor Family in Schisandra chinensis and Its Implications for secondary metabolism’ The new title accurately reflects the scope of our work without implying direct demonstration of lignan biosynthesis regulation.

Introduction-

• The introduction lacks a coherent scientific storyline. The authors should reorganize the introduction to build a stronger rationale leading to the central objective of the study.

Response: We have reorganized the Introduction into a more logical structure: (1) brief background on S. chinensis and its bioactive compounds, (2) the AP2/ERF family and its established roles in secondary metabolism, (3) the research gap and hypothesis, and (4) study objectives. This streamlined structure builds a clearer rationale leading to the study objectives.

• A substantial portion of the introduction is devoted to background information that does not directly contribute to the research objective. This results in an unnecessarily lengthy introduction and distracts the reader from the main scientific question.

Response: We have condensed the Introduction by approximately 40%, removing detailed descriptions of medicinal properties and the lignan biosynthetic pathway that did not directly contribute to the rationale for studying AP2/ERF transcription factors.

• The introduction fails to adequately justify why AP2/ERF transcription factors was chosen for investigation. A stronger mechanistic rationale supported by previous studies is needed.

Response: We have added specific examples of AP2/ERF transcription factors that regulate secondary metabolism in medicinal plants (SmERF1L1, PgERF120, Ii049), providing a stronger mechanistic rationale for focusing on this family in S. chinensis.

Materials and Methods-

• The formatting of botanical names is inconsistent throughout the manuscript. All scientific names of plant species should be written in italics. in accordance with standard taxonomic conventions. Please check and correct this throughout the manuscript.

Response: We have carefully checked and corrected all botanical names throughout the manuscript to ensure consistent italicization in accordance with taxonomic conventions.

• Please clarify the rationale for constructing the phylogenetic tree using only Arabidopsis thaliana and the study species. A phylogenetic tree based on only two species provides limited evolutionary insight and is generally insufficient to support evolutionary inferences. Inclusion of homologs from additional representative species would substantially strengthen the phylogenetic analysis.

Response: We acknowledge this limitation. The two-species comparison approach was adopted because A. thaliana provides the most thoroughly characterized AP2/ERF reference with established classification criteria, and this approach has been widely used in previous studies [1-2].

[1]Hu F, Zhang Y, Guo J. Identification and Characterization of AP2/ERF Transcription Factors in Yellow Horn. Int J Mol Sci. 2022 Nov 30;23(23) pii: ijms232314991. doi: 10.3390/ijms232314991. PubMed PMID: 36499319.

[2]Song X, Li Y, Hou X. Genome-wide analysis of the AP2/ERF transcription factor superfamily in Chinese cabbage (Brassica rapa ssp. pekinensis). BMC Genomics. 2013 Aug 23;14:573. pii: 1471-2164-14-573. doi: 10.1186/1471-2164-14-573. PubMed PMID: 23972083.

• The cis-regulatory element analysis appears to rely on conventional prediction resources. The authors are encouraged to explore and compare results with more recent and comprehensive plant cis-regulatory databases or motif resources to improve the reliability and biological relevance of the predicted regulatory elements.

Response: We used PlantCARE, which remains one of the most widely used and curated databases for plant cis-acting regulatory elements. While we acknowledge that newer resources may offer complementary information, PlantCARE provides well-validated element annotations that are suitable for comparative promoter analysis. We have maintained this approach while noting in the Discussion that the predictions represent potential regulatory mechanisms requiring experimental confirmation.

• Please justify the rationale for using Arabidopsis thaliana as the reference species for the synteny analysis.

Response: A. thaliana was chosen because it has the most extensively annotated plant reference genome, enabling robust detection of conserved syntenic blocks. This approach is consistent with previous studies on medicinal plant gene families.

• The authors have analyzed the tissue-specific expression pattern of AP2/ERF genes; however, it is unclear how these expression profiles were associated with lignan biosynthesis. Were lignan contents or compositions quantified in the corresponding tissues?

• AP2/ERF transcription factors constitute a large and functionally diverse family involved in numerous biological processes, including plant growth, development, and responses to various biotic and abiotic stresses. Therefore, tissue-specific expression alone is insufficient to infer their specific role in lignan biosynthesis.

Response: We thank the reviewer for this important question. We agree that associating gene expression profiles with lignan biosynthesis requires quantitative data on lignan content in the corresponding tissues. In this study, we did quantify lignan accumulation across different tissues of S. chinensis.

Specifically, we determined the content of schisandrol A, the quality marker component of S. chinensis according to the Chinese Pharmacopoeia, in roots, stems, leaves, and fruits using HPLC. The results are presented in Fig 8A–B, which show a clear tissue-specific accumulation pattern: fruits > stems > roots > leaves. This trend is consistent with previous reports that lignans are primarily enriched in fruits.

We then compared this tissue-specific lignan distribution with the expression profiles of the ten fruit-preferential ScAP2/ERF genes. Notably, these ten genes exhibited fruit-specific or fruit-preferential high expression, which coincides with the highest accumulation of schisandrol A in fruits. This spatial correlation suggests that these ScAP2/ERF genes may be involved in regulating lignan biosynthesis in the medicinal fruit of S. chinensis. In addition, qRT-PCR validation confirmed that the expression patterns of these ten genes were consistent with the RNA-seq data, further supporting the reliability of our expression analysis.

We acknowledge that we only quantified schisandrol A as a representative lignan, rather than the full spectrum of lignan compounds. This is a limitation of the current study.

• The qRT-PCR validation appears to be based only on biological replicates. Please clarify whether technical replicates were performed.

Response: We have clarified in the revised Methods that three technical replicates were performed for each of the three biological replicates.

• The manuscript does not specify the statistical methods used to analyze the qRT-PCR data. Please clearly describe the statistical software, tests employed and significance thresholds used for the analysis.

Response: We have added a detailed description of the statistical methods in the revised Methods section, specifying one-way ANOVA followed by Tukey’s HSD test, implemented in SPSS Version 26.0. Significance threshold (P < 0.05) and data presentation format (mean ± SE) have also been specified.

Results-

• The subcellular localization of the candidate protein(s) has been predicted only through computational approaches. Experimental validation, such as transient expression of GFP fusion constructs in Nicotiana benthamiana leaves, would substantially strengthen the results.

Response: We appreciate this suggestion. While experimental validation through GFP fusion constructs would indeed strengthen the results, this is beyond the scope of the current genome-wide identification study. We have acknowledged this limitation in the Discussion, noting that ‘the subcellular localization of ScAP2/ERF proteins was predicted computationally and has not been experimentally verified.’ This represents an important direction for future research.

• Figure 2 is difficult to interpret as the multiple sequence alignment is not organized according to the AP2/ERF subfamilies identified in the phylogenetic tree. To improve clarity authors should rearrange the alignment by grouping sequences according to their respective subfamilies.

Response: We sincerely thank the reviewer for this constructive suggestion. We fully agree that organizing the multiple sequence alignment according to the AP2/ERF subfamilies identified in the phylogenetic tree is essential for clearly presenting subfamily-specific sequence features. In the revised manuscript, we have completely rearranged the sequence order in Figure 2 based strictly on the phylogenetic analysis results. Sequences are now grouped and displayed according to their respective subfamilies, with members of the same subfamily placed adjacently. This arrangement allows readers to intuitively compare conserved motifs and key amino acid residues across different subfamilies.

• Tissue-specific expression analysis is a key component of this study; however, no representative images of the plant tissues used for qRT-PCR are provided. The authors are encouraged to include photographs of the sampled tissues, either in the main manuscript or as supplementary material, to clearly document the tissue sampling strategy and enhance the reproducibility of the study.

Response:

We thank the reviewer for this valuable suggestion. To improve the clarity and reproducibility of our tissue sampling strategy, we have now included representative photographs of the plant tissues used for qRT-PCR analysis (root, stem, leaf, and fruit) in Fig 7A of the revised manuscript.

Discussion-

• The manuscript requires extensive English language editing. Numerous grammatical mistakes, awkward phrasing (eg. his research conducted a comprehensive genome-wide identification), inconsistent tense usage, and poor sentence construction are present throughout the manuscript, making several sections difficult to read.

• The authors extensively discuss the regulatory roles of MeJA and ABA despite the absence of any experimental validation, such as hormone treatment assays or expression analyses following MeJA/ABA application. Since cis-regulatory element prediction only suggests potential regulatory mechanisms, the current discussion appears speculative and should be revised accordingly.

Response: We sincerely thank the reviewer for this constructive criticism. We fully agree that cis-regulatory element prediction alone cannot establish regulatory roles, and that our original discussion of MeJA and ABA was overly speculative. To address this concern, we have performed additional experiments and revised the manuscript accordingly.

We performed MeJA treatment on S. chinensis seedlings and examined the expression of the ten fruit-preferential ScAP2/ERF genes. The detailed method has been added to the Materials and Methods section:

S.chinensis seedlings at a height of 20-30 cm were selected for hormone treatment. A 1 mM methyl jasmonate (MeJA) solution was prepared and applied to the plants using two routes: 100 mL was sprayed onto the foliar surface, and another 100 mL was drenched into the soil around the roots. Leaf tissues were collected at 0, 6, 12, 24, and 48 h post-treatment. Immediately after harvesting, the leaves were rinsed thoroughly with distilled water, blotted dry, and flash-frozen in liquid nitrogen. All samples were stored at -80℃ until used for qRT-PCR analysis. Three independent biological replicates were performed for each time point.

The expression results of the ten fruit-preferentially expressed ScERFs are shown in Fig 8D. Consistent with our promoter prediction, all ten genes responded to MeJA, with ScERF38, ScERF100, ScERF104, ScERF107, and ScERF108 peaking at 6 h, and ScERF05, ScERF40, ScERF64, and ScERF102 peaking at 12 h. These data provide direct experimental evidence for MeJA responsiveness and support the potential involvement of these ScAP2/ERF genes in MeJA-mediated regulation.

• The last paragraph of the Discussion is largely repetitive and overlaps substantially with the Conclusion section. It should be removed or significantly shortened to eliminate redundancy and improve the manuscript's readability.

Response: We have removed the repetitive content from the last paragraph of the Discussion. The Discussion now ends with the limitations paragraph, while the Conclusion section provides a concise summary of the main findings.

Several references are not formatted according to the journal's guidelines. Please carefully check and correct the formatting of all references to ensure consistency throughout the reference list.

The figure legends should be more comprehensive. They should include sufficient methodological and explanatory details so that each figure can be understood without referring extensively to the main text.

Response: We have carefully reviewed and reformatted all references to ensure consistency with PLOS ONE guidelines. We have enhanced all figure legends to include sufficient methodological and explanatory details so that each figure can be understood without extensive reference to the main text.

We hope that these revisions satisfactorily address all the concerns raised by the reviewers. We believe that the manuscript has been substantially improved and is now suitable for publication in PLOS ONE.

Sincerely,

Wei Nie (on behalf of all authors)

Nanjing Research Institute for Comprehensive Utilization of Wild Plants

niewei@co-plant.com

Attachment Submitted filename: Response to Reviewers.docx

# Peer review: Decision Letter 1

**DOI:** 10.1371/journal.pone.0361017.r003

**Authors:** Anil Kumar Singh

6 Oct 2026

<p>Comprehensive Analysis of the AP2/ERF Transcription Factor Family in Schisandra chinensis and Its Implications for Lignan Biosynthesis

PONE-D-26-31522R1

Dear Dr. NIE,

We’re pleased to inform you that your manuscript has been judged scientifically suitable for publication and will be formally accepted for publication once it meets all outstanding technical requirements.

Within one week, you’ll receive an e-mail detailing the required amendments. When these have been addressed, you’ll receive a formal acceptance letter and your manuscript will be scheduled for publication.

An invoice will be generated when your article is formally accepted. Please note, if your institution has a publishing partnership with PLOS and your article meets the relevant criteria, all or part of your publication costs will be covered. Please make sure your user information is up-to-date by logging into Editorial Manager at Editorial Manager® and clicking the ‘Update My Information' link at the top of the page. For questions related to billing, please contact billing support.

If your institution or institutions have a press office, please notify them about your upcoming paper to help maximize its impact. If they’ll be preparing press materials, please inform our press team as soon as possible -- no later than 48 hours after receiving the formal acceptance. Your manuscript will remain under strict press embargo until 2 pm Eastern Time on the date of publication. For more information, please contact onepress@plos.org.

Kind regards,

Anil Kumar Singh, Ph.D.

Academic Editor

PLOS One

Additional Editor Comments (optional):

Reviewers' comments:

Reviewer's Responses to Questions

**Comments to the Author**

Reviewer #1: All comments have been addressed

Reviewer #2: All comments have been addressed

**********

2. Is the manuscript technically sound, and do the data support the conclusions?

Reviewer #1: Yes

Reviewer #2: Yes

**********

3. Has the statistical analysis been performed appropriately and rigorously?

Reviewer #1: Yes

Reviewer #2: Yes

**********

4. Have the authors made all data underlying the findings in their manuscript fully available?

The PLOS Data policy

Reviewer #1: Yes

Reviewer #2: Yes

**********

5. Is the manuscript presented in an intelligible fashion and written in standard English?

Reviewer #1: Yes

Reviewer #2: Yes

**********

Reviewer #1: Dear Authors

All comments have been adressed. I recommend the acceptance for this research.

With the best wishes,

Reviewer #2: (No Response)

**********

what does this mean?). If published, this will include your full peer review and any attached files.

If you choose “no”, your identity will remain anonymous but your review may still be made public.

**Do you want your identity to be public for this peer review?**  For information about this choice, including consent withdrawal, please see our Privacy Policy

Reviewer #1: **Yes:**  ALI MOGHADAM

Reviewer #2: No

**********

# Editor report: Acceptance letter

**DOI:** 10.1371/journal.pone.0361017.r004

**Authors:** Anil Kumar Singh

PONE-D-26-31522R1

PLOS One

Dear Dr. NIE,

I'm pleased to inform you that your manuscript has been deemed suitable for publication in PLOS One. Congratulations! Your manuscript is now being handed over to our production team.

At this stage, our production department will prepare your paper for publication. This includes ensuring the following:

* All references, tables, and figures are properly cited

* All relevant supporting information is included in the manuscript submission,

* There are no issues that prevent the paper from being properly typeset

You will receive further instructions from the production team, including instructions on how to review your proof when it is ready. Please keep in mind that we are working through a large volume of accepted articles, so please give us a few days to review your paper and let you know the next and final steps.

Lastly, if your institution or institutions have a press office, please let them know about your upcoming paper now to help maximize its impact. If they'll be preparing press materials, please inform our press team within the next 48 hours. Your manuscript will remain under strict press embargo until 2 pm Eastern Time on the date of publication. For more information, please contact onepress@plos.org.

You will receive an invoice from PLOS for your publication fee after your manuscript has reached the completed accept phase. If you receive an email requesting payment before acceptance or for any other service, this may be a phishing scheme. Learn how to identify phishing emails and protect your accounts at https://explore.plos.org/phishing.

If we can help with anything else, please email us at customercare@plos.org.

Thank you for submitting your work to PLOS One and supporting open access.

Kind regards,

PLOS One Editorial Office Staff

on behalf of

Dr. Anil Kumar Singh

Academic Editor

PLOS One
